# Early Incorporation to Palliative Care (EPC) in Patients With Advanced Non-Small Cell Lung Cancer: The PACO Randomized Clinical Trial

**DOI:** 10.1093/oncolo/oyae050

**Published:** 2024-04-01

**Authors:** Silvia Allende, Jenny G Turcott, Emma Verástegui, Oscar Rodríguez-Mayoral, Diana Flores-Estrada, Dana Aline Pérez Camargo, Maritza Ramos-Ramírez, Jorge-Negueb Martínez-Hernández, Luis F Oñate-Ocaña, Pamela Soberanis Pina, Andrés F Cardona, Oscar Arrieta

**Affiliations:** Department of Palliative Care, Instituto Nacional de Cancerología, Mexico City, México; Thoracic Oncology Unit, Instituto Nacional de Cancerología, Mexico City, México; Department of Palliative Care, Instituto Nacional de Cancerología, Mexico City, México; Department of Palliative Care, Instituto Nacional de Cancerología, Mexico City, México; Thoracic Oncology Unit, Instituto Nacional de Cancerología, Mexico City, México; Department of Palliative Care, Instituto Nacional de Cancerología, Mexico City, México; Thoracic Oncology Unit, Instituto Nacional de Cancerología, Mexico City, México; Comprehensive Cancer Center, Medica Sur Foundation and Clinic, Mexico City, México; Thoracic Oncology Unit, Instituto Nacional de Cancerología, Mexico City, México; Thoracic Oncology Unit, Instituto Nacional de Cancerología, Mexico City, México; Research and Education Direction, Luis Carlos Sarmiento Angulo Cancer Treatment and Research Center (CTIC), Bogotá, Colombia; Foundation for Clinical and Applied Cancer Research, FICMAC and Molecular Oncology and Biology Systems Group, Universidad El Bosque, Bogotá, Colombia; Thoracic Oncology Unit, Instituto Nacional de Cancerología, Mexico City, México

**Keywords:** palliative care, depression, anxiety, survival, quality of life

## Abstract

**Background:**

Patients with non-small cell lung cancer (NSCLC) experience a considerable disease burden, evident in symptomatic and psychological spheres. Advanced cancer represents a complex scenario for patients and the healthcare team. Early palliative care (EPC) has been proven as a clinically meaningful strategy in this context by several randomized trials but not in a resource-limited setting. This study aimed to evaluate the effect of EPC compared with standard oncological care (SOC) in patients with metastatic NSCLC in Mexico.

**Materials and Methods:**

A prospective, randomized clinical trial was conducted at Instituto Nacional de Cancerologia in Mexico. All patients had histologically confirmed metastatic NSCLC without previous treatment. Patients were randomly assigned (1:1) to receive SOC or SOC + EPC. The EPC group was introduced to the palliative care team at baseline after randomization, which was integrated by psychologists, bachelor’s in nutrition, specialized nurses, and physicians. Patients randomized to this arm had programmed visits to meet with the team at baseline and through the 2nd, 4th-, and 6th cycles thereafter. The primary endpoint was overall survival (OS); secondary outcomes included quality of life (QoL), anxiety and depression, and symptom intensity. They were assessed using the instruments EORTC QLQ-C30 questionnaire, Edmonton Symptom Assessment Scale (ESAS), and the Hospital Anxiety and Depression Scale (HADS) (clinicaltrials.gov [NCT01631565]). Questionnaires were completed at baseline, at 2nd, 4th, and 6^th^ cycles of treatment.

**Results:**

Between March 2012 and June 2015, 201 patients were assessed for eligibility and 146 were enrolled and allocated to receive EPC (73) or SOC (73). Median OS for patients in the EPC vs SOC arm was 18.1 months (95% CI, 7.9-28.4) and 10.5 months (95% CI, 4.7-16.2) (*P* = .029). Having a poor performance status (HR 1.7 [1.2-2.5]; *P* = .004) and allocation to the control group (HR 1.5 [1.03-2.3]; *P* = .034) were independently associated with a worse OS. Those patients with a global QoL > 70 at baseline had a better OS if they were In the EPC arm (38.7 months (95% CI, 9.9-67.6) vs SOC 21.4 months (95% CI, 12.4-30.3)). Mean QoL had a numerical improvement in patients allocated to EPC after 6 cycles of follow-up, nonetheless this difference was not statistically significant (55.1 ± 23.7 vs 56.9 ± 25.3; *P* = .753). There were no significant differences in anxiety and depression at all study points.

**Conclusions:**

EPC is associated with a significant improvement in OS, although, we observed that the greatest benefit of providing EPC was observed in those with a global QoL > 70 at baseline. This study did not identify significant changes in terms of QoL or symptom burden between the study groups after follow-up. Evidence robustly suggests that EPC should be considered part of the multidisciplinary treatment of metastatic NSCLC patients since diagnosis. According to our study, EPC can be implemented in low- or middle-income countries (LMIC).

## Teaser

The benefit of early palliative care in a resource-limited setting is not well understood. This study examined whether early palliative care could improve the survival of Mexican patients with advanced non-small cell lung cancer compared with standard oncological care.

**Implications for Practice**

Early palliative care (EPC) interventions are recommended in oncological practice guidelines due to significant benefits in clinical outcomes, especially in quality of life and symptom intensity. Overall survival may have a beneficial effect; however, it has not been the primary endpoint in pivotal studies assessing EPC. This is the first study conducted in Latin America to evaluate the efficacy of EPC in terms of overall survival as the primary endpoint and most importantly validates the finding of other studies in a resource-limited setting. This study highlights that EPC is a relevant and feasible strategy to implement in resource-limited settings and should be offered to all patients who agree to the intervention as it may confer significant benefits in survival outcomes.

## Introduction

Lung cancer is a major global public health problem, with approximately 2.2 million new cases yearly and currently leading the cancer-related mortality list^1-3^ Moreover, patients with NSCLC face a significant burden in terms of symptoms, QoL and financial strain,^4^ particularly for those diagnosed in the III or IV stage setting,^5^ which unfortunately represents most of the cases worldwide.

Despite recent advances in cancer therapeutics, the current 5-year survival rate for patients diagnosed with lung cancer ranges from 10% to 20%, the lowest among the most common neoplasms.^6-8^ Patients with advanced cancer (stage III or IV) need a special focus on patient-centered care.^9-20^ Aggressive treatments for metastatic NSCLC can cause significant morbidity, increase symptom burden, and dramatically augment treatment costs without significant impact on survival outcomes.^9-20^ Currently, there seems to be a positive shift in the quality of EOL care focusing on supportive care. The timing of the palliative care consultation can attenuate the delivery of aggressive interventions.^9-20^

Presently, international guidelines recommend that patients with advanced cancer be offered early palliative care (EPC) along with standard oncological care (SOC).^21^ Evidence from this recommendation stems from a previously published randomized clinical trial which demonstrated a significant benefit in terms of QoL and overall survival (OS), in addition to reduction in aggressive EOL treatment^9,10^ as well as, from other studies that have consistently demonstrated the benefits of EPC interventions.^22-24^ EPC has been shown to be feasible to implement and to be a cost-effective intervention that can decrease the burden of disease for patients with NSCLC.^25,26^ Altogether, EPC plays a key role in terms of the medical care of these patients, focusing on psychological support, symptom management, and facilitation in decision-making.

However, in Latin America (LA) it is usually offered to patients only after life-prolonging treatment has failed. Available prospective literature regarding EPC in low- and low-middle-income countries (LMIC) is more limited, where EPC seldom happens and can be challenging due to access, lack of sufficient trained healthcare providers, sociocultural factors, and financial constraints.^27-31^ In line with this, a very recent publication has highlighted the discrepancy between the ideal and actual timing of palliative care for children with cancer in LA. They conducted a survey among physicians in 17 countries in LA and found that physicians believe that palliative care should be integrated early in cancer care, yet they recognize that this does not occur in the clinic and multiple barriers have to be overcome,^32^ which reflects a similar situation in adult patients with cancer. In Mexico, less than a third of the patients with a recent diagnosis of advanced cancer (less than 1 year) are early referred to palliative care.^31^ Further investigations are needed to urge the incorporation of this practice throughout cancer centers in LMIC.^27^ In addition, current evidence shows that patients with metastatic cancer who receive EPC have a significant improvement in QoL and mood.^10,26^ Hence, the rationale of this trial was to examine whether EPC could improve the survival of Mexican patients with advanced NSCLC compared with SOC. We hypothesized that the incorporation of EPC could be feasible in a LMIC and that patients with advanced NSCLC who receive it could improve their survival. The results of this trial would help to provide additional prospective evidence regarding the benefits of EPC in Latin America.

## Materials and Methods

### Study Design and Participants

This was a single-center, randomized, non-blinded, parallel-group controlled trial. Patients from the Thoracic Oncology Unit at the Instituto Nacional de Cancerología (INCan) in Mexico were invited to participate in the study. Eligible patients had pathologically confirmed metastatic NSCLC without previous treatment and were diagnosed within the previous 8 weeks and were able to read and answer questions in Spanish. Patients who were already attending the palliative care clinic were excluded. All participants gave written informed consent. This study was approved by the local Institutional Review Board and Ethics Committee (013/020/ICI)(CV773/13). The study is registered on clinicaltrials.gov (NCT01631565) (Supplementary Fig. S1).

### Randomization and Blinding

Eligible patients who agreed to participate were randomized (1:1) using a random numbers table and allocated to the control arm, which received SOC alone, or the experimental arm, which received SOC plus EPC. Patient randomization was not masked, so the patient and healthcare provider were aware of the assignment. QoL questionnaires were answered as part of the follow-up.

### Intervention and Assessments

EPC offered care for patients and their families who were facing a life-limiting illness (metastatic lung cancer). It involved combining palliative support with standard of care after a patient was diagnosed with lung cancer. This approach aimed to offer a patient-centered coordinated care to relieve suffering, support understanding of the disease and coping, symptoms and function, and advanced care planning. Patients assigned to the EPC group were introduced to the palliative care team after randomization mainly immediately or with an early appointment, which was integrated by psychologists, bachelors in nutrition, specialized nurses, and physicians, all of them were board-certified and received training in terms of palliative care for patients with cancer. Patients randomized to this arm of the trial had programmed visits to meet with the palliative, nutrition, and psychological care specialists at baseline and after the 2nd, 4th, and 6th cycles. Additional visits could be scheduled at the request of the patient, their caregiver, or the attending physician. This protocol was adapted from previously published trials and guidelines and placed particular emphasis on physical, nutritional, oncologic, and psychological symptoms.^10,33^ The palliative team documented as usual their clinical notes using the electronic records at INCan (INCanet). In addition to this protocol, the palliative team was trained to discuss goals of care with the patients thoroughly, assist with decision-making regarding treatment, and discuss EOL care and options if required.

Patients allocated to receive SOC had a baseline evaluation and received it at the time of the study. They were referred to palliative only when expressly requested by the patients, their primary caregiver, or their attending physician, similar to a previous study in this population.^10^ As such, the frequency varied according to each patient’s necessity and only if requested. Participants in this group referred to the palliative care service received the same care as patients in the intervention group but did not have the same standardized follow-up.

### Assessments

Evaluation of QoL, anxiety and depression, and oncologic symptoms were conducted. Evaluation of health QoL (HRQL) was measured using the validated Mexican-Spanish version of the EORTC-European Organization for the Research and Treatment of Cancer Quality of Life Questionnaires specific for cancer and for LC (EORTC-QLQ-C30 and QLQ-LC13, respectively) which evaluates the physical, functional, emotional, social, and lung symptom status.^34,35^ Scores for functional and symptoms scales were calculated using a linear transformation of raw scores to produce a range from 0 to 100, as described by EORTC. In this scale, the best score is 100 for the global health status and functional scales, while scores nearing 0 represent lesser symptoms. The Edmonton Symptom Assessment Scale (ESAS) was used to assess symptom presence and severity perceived with a spectrum of intensity 0–10, in each visit.^36-38^ Furthermore, the Mexican population validated version^39^ of the Hospital Anxiety and Depression Scale (HADS) was used to screen anxiety and depression. Anxiety or depression: normal 0-5, light 6-8, moderate 9-11, and severe >12.

Caregiver burden was assessed using the validated version of the Zarit scale.^40^ It consists of 22 items with a Likert-type response. With an answer range from 1 (never) and 5 (almost always). The score range of this instrument is between 22 and 110 points, the higher the score the higher the perceived burden. It is considered without burden: 22-46, mild burden: 47-55, and severe burden: 56-110. Every caregiver was consulted to participate and if they were in agreement they were included in the study. Exclusion criteria were considered as the caregiver refused to participate and that the companion was not a primary caregiver or not close to the patient. Questionnaires were completed at baseline, at 2nd, 4th, and 6th cycles of treatment of their patients.

Baseline survey assessments were given after patients were informed of their randomization assignment. All data regarding the numbers, timing, chemotherapy regimens, anthropometric values, and performance status at each visit, in accordance with the study protocol, were recorded.

The patients randomized to receive EPC, were seen at the palliative care unit; after a complete evaluation, a detailed description of the recommendations was given written if required. Pharmacological treatment was thoroughly explained for each symptom present if required.

The primary endpoint was overall survival (OS). Secondary endpoints included QoL, depression, anxiety, caregiver burden, and symptom burden difference between study groups and through follow-up.

### Sample Size

The sample size was calculated based on the 2-year survival rate reported in previous trials assessing EPC,^10^ to detect a difference of 23%. Power was set at 0.80 and significance at 0.05 with a 2-tailed test, and for a minimum sample of 123 patients.

### Statistical Analysis

Continuous data were summarized as arithmetic means with SDs or medians (percentile 25-75) according to data distribution. Mean or median comparisons between groups were performed using a *t*-test or Mann–Whitney *U* test, for paired comparisons we used a paired *T*-test and Wilcoxon test. Categorical variables were summarized as frequencies and percentages, and comparisons among them were performed using the chi-squared test and for categories with less than 5 events comparisons were made using a Fisher´s exact test. Further, for continuous scales (eg, QoL and symptomatic burden) we estimated the gross mean change in the score along with 95% confidence intervals (CI) to assess the changes in those scales from baseline compared with at 6 cycles post-EPC start. Paired comparisons were performed using paired *t*-tests. OS was estimated from the date of diagnosis until death or last follow-up, using the Kaplan-Meier method, while comparisons among groups were analyzed with log-rank tests. A multivariate analysis using Cox proportional hazard was performed for patients. For the multivariate analyses following the baseline analysis, data was censored according to the number of patients who were enrolled at that specific time point. Statistical significance was determined preferentially with the 95% CI, *P* ≤ .05 was also obtained; 2-sided tests were used in all cases. All data were analyzed using the SPSS software package version 20 (SPSS, Inc., Chicago, IL, USA).

## Results

### Study Population

**Figure 1.**
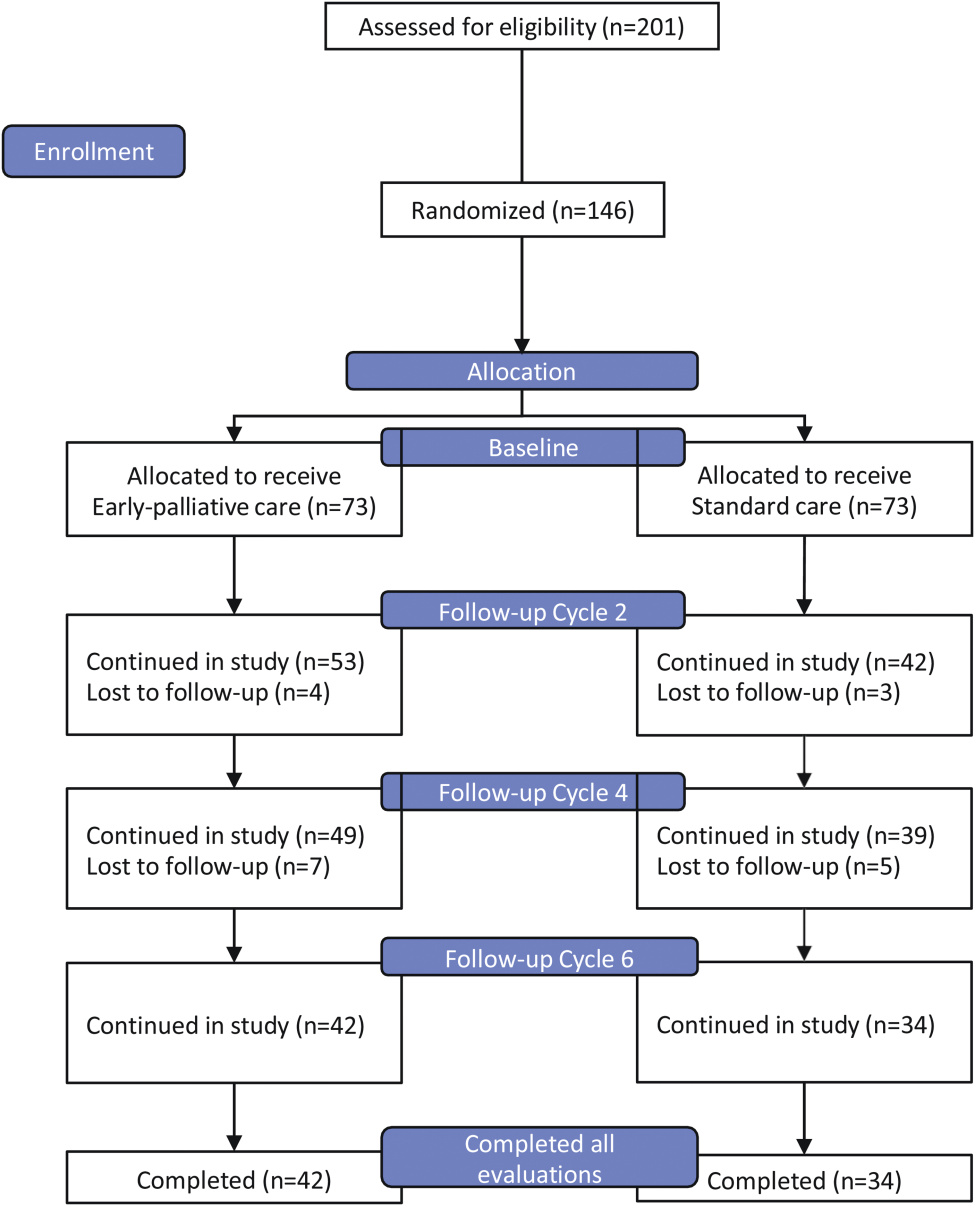
CONSORT 2010 flow diagram.

A total of 201 patients were evaluated for eligibility, 55 patients were excluded due to inability to show up to clinic appointments or complete follow-up (not able to commit to schedule due to distance, lack of social/family support, or limited financial resources to travel frequently). A total of 146 patients met the inclusion criteria and agreed to participate in the study. Among these, 73 (50%) were allocated to receive EPC and 73 (50%) were assigned to receive SOC. Patients were evaluated at baseline and followed at the end of the 2nd, 4th, and 6th cycle of therapy for a minimum of 4 visits with the EPC team. During follow-up, 31 patients in the EPC arm failed to complete study visits or questionnaires, died or were lost to follow up, leaving a total of 42 patients at the end of the 4th evaluation. In comparison, 39 patients failed to complete study visits or questionnaires, died, or were lost during follow-up on the SOC arm, leaving 34 patients at the end of the 4th evaluation (Fig. 1).

Baseline characteristics were well-balanced between both arms of the trial following randomization. Detailed information regarding the study population is summarized in Table 1.

**Table 1.** Baseline characteristics of study participants.

Variable	Standard care plus early palliative care *N* = 73	Standard care alone *N* = 73	*P*-value
Sex			
Female	37 (50.7)	32 (43.8)	0.407
Male	36 (49.3)	41 (56.2)	
Age (years)			
Mean (±SD)	59.5 ± 12.6	61.6 ± 14.05	0.327
<60	38 (52.1)	32 (43.8)	0.320
≧60	35 (47.9)	41 (56.2)	
Tobacco exposure			
Present	42 (57.5)	39 (53.4)	0.617
Absent	31 (42.5)	34 (46.6)	
Wood smoke exposure			
Present	29 (39.7)	24 (32.9)	0.394
Absent	43 (58.9)	49 (67.1)	
Unknown	1 (1.4)	0 (0)	
ECOG performance status			
0–1	60 (82.2)	56 (76.7)	0.413
>1	13 (17.8)	17 (23.3)	
Initial anticancer therapy			
No chemotherapy	3 (4.1)	9 (12.4)	0.068
Cis/carboplatin/taxane	57 (78.1)	59 (80.8)	
Cis/carboplatin/pemetrexed	8 (11.0)	2 (2.7)	
Cis/carboplatin/gemcitabine	5 (6.8)	3 (4.1)	
TKI	0 (0)	0 (0)	
Other	0 (0)	0 (0)	
*EGFR* mutations			
* EGFR* mutation	14 (19.2)	8 (11.0)	0.116
* EGFR-*wild type	28 (38.3)	22 (30.1)	
Unknown	31 (42.5)	43 (58.9)	
Median QoL at baseline			
Global status	50 (33.33-77.08)	50 (33.33-66.67)	0.701

Abbreviations: ECOG, Eastern Cooperative Oncology Group; EGFR, epidermal growth factor receptor; QoL, Quality of life.

### Adherence to the Intervention

Following randomization, a total of 95.9% (*N* = 70/73) of patients in the EPC arm of the trial completed the baseline evaluations, compared with 91.8% (*N* = 67/73) patients in the control arm. Among patients allocated to the EPC arm of the study, *n* = 53 completed at least one follow-up visit and evaluation before the 2nd chemotherapy cycle; moreover, a total of *n* = 42 completed all the study follow-up visits and assessments and have data from baseline to the 6th cycle. In the control arm of the study, *n* = 42 completed the follow-up visit and evaluation before the 2nd chemotherapy cycle, and *n* = 34 patients completed all follow-up visits and assessments from baseline until the 6th cycle. At the time of the final analysis, a total of 100 deaths had occurred, among which 46 were recorded among patients allocated to the EPC arm of the trial and 54 were recorded among patients allocated to the SOC arm of the study.

### Overall Survival

**Figure 2.**
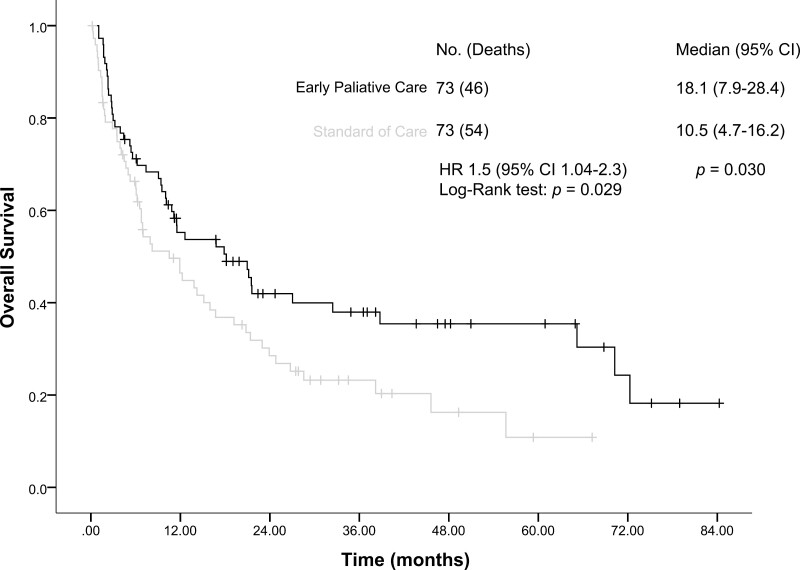
Kaplan-Meier analysis of overall survival (OS) for the entire study population.

The median follow-up was 10.9 months (95% CI, 3.8-26.8) for the entire study population. In terms of the primary outcome, patients in the EPC arm of the trial had a significantly longer median OS compared with patients allocated to the SOC arm (18.1 months [95% CI, 7.9-28.4] vs 10.5 months [95% CI, 4.7-16.2]; HR: 1.5 [95% CI, 1.04-2.3]; *P* = .030; Fig. 2). Mortality rate in EPC group at 6 months was 29% compared to 33% in SOC group and after 12 months 44% in EPC group, and 53% in SOC group. In the multivariate analysis, having an ECOG performance status of > 1 (HR: 1.7 [95% CI, 1.2-2.5]; *P* = .004) and being allocated to the control arm of the study (HR: 1.5 [95% CI, 1.03-2.3]; *P* = .034) were independently associated with worse OS for the entire study population (Table 2). However, when analyzing the survival of patients who did not receive treatment, the median was 1.1 months (95% CI, 1.0-1.1), 3 patients from the EPC group and 9 patients from control group. Cox regression analysis including good performance status ECOG of ≤1 (HR: 0.6 [95% CI, 0.4-1.0]; *P* = .043) and those on treatment (HR: 0.7 [95% CI, 0.5-0.9], *P* = .020) showed that these factors were independently associated with greater survival in this group of patients. Of note, being allocated to the EPC arm (HR: 0.7 [95% CI, 0.5-1.0]; *P* = .075) showed a trend in survival, although not statistically significant.

**Table 2.** Univariate and multivariate analysis of factors associated with overall survival.

Variables	*N* (deaths)	Median (95% CI)	*P*	Univariate HR (IC 95%)	*P*	Multivariate HR (95%CI)	*P*
Overall survival	146 (100)	12.6 (7.8-17.4)					
Age (years)							
<60	70 (42)	19.1 (13.8-24.5)	**0.066**	1.4 (0.9-2.1)	0.067		
≧60	76 (58)	10.0 (4.7-15.2)					
Sex							
Female	69 (41)	20.7 (10.3-31.2)	0.074	1.4 (0.9-2.1)	0.076		
Male	77 (59)	10.1 (3.07-17.1)					
ECOG							
0-1	116 (77)	16.7 (9.3-24.1)	**0.013**	1.7(1.1-2.8)	**0.015**	1.7 (1.2-2.5)	**0.004**
>1	30 (23)	6.7 (5.6-7.9)					
Tobacco exposure							
Absent	65 (43)	12.6 (2.1-23.0)	0.738	1.07 (0.7-1.5)	0.739		
Present	81 (57)	12.2 (6.4-18.0)					
Wood-smoke exposure							
Absent	92 (66)	11.4 (6.5-16.4)	0.832	0.9 (0.6-1.4)	0.832		
Present	53 (34)	15.9 (8.-23.7					
Asbestos exposure							
Absent	133 (89)	14.2 (1.9-74.3)	0.454	1.5 (0.8-2.9)	0.160		
Present	13 (11)	55.6 (8.1 -103)					
*EFGR* status							
* EGFR-*wild type	50 (24)	38.1 (1.9 -74.3)	0.000	1.8 (1.4-2.3)	0.000		
* EGFR-*mutated	22 (11)	55.6 (8.1-103.1)					
Unknown	74 (65)	6.2 (4.4-8.0)					
Adenocarcinoma histology							
Negative	18 (11)	21.5 (9.9-33.0)	0.232	0.6 (0.3-1.2)	0.235		
Positive	128 (89)	11.9 (6.8 - 17)					
Albumin g/dl							
< 3.5	69 (53)	6.9 (2.06-11.8)	**0.000**	2.0 (1.4-3.0)	**0.001**		
3.5+	75 (46)	21.5 (9.3-33.7)					
Unknown	2 (1)	1.7 (NR)					
Group							
Early palliative care	73 (46)	18.1 (7.9-28.4)	**0.029**	1.6 (1.04-2.3)	**0.030**	1.5 (1.03-2.3)	**0.034**
Standard of care	73 (54)	10.5 (4.7-16.2)					
Depression at baseline							
Absent	69 (48)	20.7 (14.5-27.0)	**0.000**	1.0 (0.8-1.3)	**0.939**		
Present	54 (47)	6.0 (4.5-7.6)					
Unknown	23 (5)	NR					
Anxiety at baseline							
Absent	61 (45)	15.1 (5.6-24.6)	0.003	0.8 (0.7-1.2)	0.329		
Present	62 (50)	6.7 (4.7-8.7)					
Unknown	23 (5)	NR					
QoL at baseline							
> 70	38 (23)	27.0 (9.0-45.0)	0.000	2.6 (1.7-4.1)	**0.000**		
≦** 70**	99 (68)	11.1 (6.5-15.8)					
Unknown	9 (9)	3.4 (1.5-1.8)					

Abbreviations: ECOG, Eastern Cooperative Oncology Group; EGFR, epidermal growth factor receptor; QoL, quality of life.

**Figure 3.**
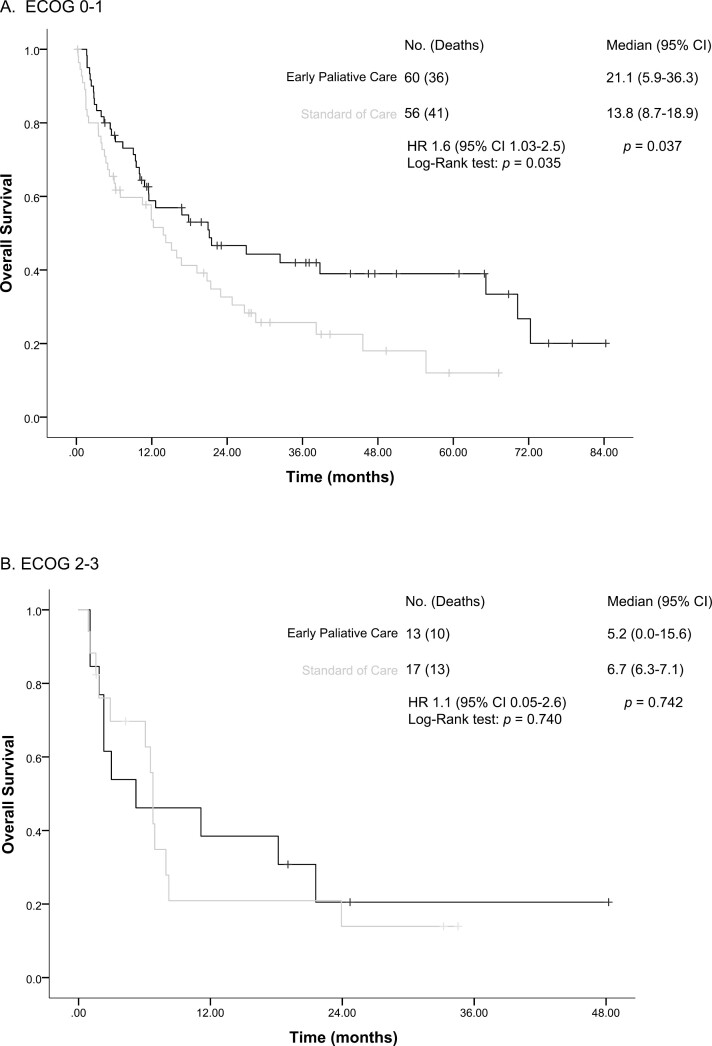
Kaplan-Meier analysis of overall survival between groups according to performance status: (**a**) overall survival among patients with good performance (ECOG 0-1) and (**b**) overall survival among patients with ECOG 2-3.

We performed a post hoc sub-analysis stratifying patients according to ECOG performance status (ECOG 0-1 vs >1) and study allocation. Results showed that patients with a good performance status (ECOG 0-1) had a significantly improved OS when allocated to EPC compared with those assigned to receive SOC (21.1 months [95% CI, 5.9-36.3] vs 13.8 months [95% CI, 8.7-18.9]; HR: 1.6 [95% CI, 1.03-2.5] *P* = .037; Fig. 3).

A second post hoc analysis stratifying patients according to Global QoL (GQoL ≤ 70 vs >70) and study allocation. We observed a significantly better OS in those with GQoL > 70 in the EPC arm (38.7 months [95% CI, 9.9-67.6]) compared to SOC (21.4 months [95% CI, 12.4-30.3], *P* = .048). Comparing study groups in those with low global QoL ≤ 70 no significant differences were observed in terms of OS (EPC 11.5 [95% CI, 3.3-19.8] vs SOC 8.0 months [95% CI, 1.7-14.2], *P* = .325; Supplementary Fig. S2).

### Quality of Life

At baseline, patients in the EPC arm of the study had a numerically higher score in terms of mean global health status, although this was not statistically significant (56.9 [±25.4] vs 48.5 [±26.6]; *P* = .061). QoL remained without significant differences when contrasting the mean difference at the evaluations performed at 6 cycles (−8.1 [95% CI, −21.0, 4.7]; *P* = .213; Table 3). The mean difference for each of the subscales in the functional QoL evaluation was also comparable from baseline to the 6th cycle assessment, and we did not identify significant differences in terms of physical functioning (mean difference of −6.0 [95% CI: −18.1, 6.1]; *P* = .329), role functioning (mean difference of 6.4 [95% CI, −9.6, 22.5]; *P* = .426), emotional functioning (mean differences of −38 [95% CI, −15.3, 7.5, *P* = .501), cognitive functioning (mean difference of 3.5 [95% CI, −10.3, 17.4]; *P* = .615), or social functioning (mean difference of −5.8 [95% CI, −23.8, 12.1]; *P* = .522). The evaluation of pain showed that patients allocated to the control arm of the trial had a mean difference of 6.8 (±27.8) when contrasting baseline and 6th cycle evaluation. In contrast, patients in the experimental arm had a non-significant improvement (−4.3 ± 31.6) (mean difference in control and experimental groups −-11.2 [95% CI, −25.0, 2.5]*; P* = .109). In a post hoc sub-analysis, patients were stratified according to ECOG performance; however, the mean difference in global health status when comparing patients with a good performance status (ECOG 0-1) allocated to the control and experimental groups was also non-significant (−11.4 [−25.1 to 2.2]; *P* = .100) (Supplementary Tables S1, S2). Further, when stratifying patients by depression, we identified that patients with a baseline diagnosis of depression allocated to the EPC arm of the study had a significant improvement in pain, with a mean difference of −29.6 (95% CI, −58.7, 0.5); *P* = .046 (Supplementary Tables S3, S4).

**Table 3.** Main quality-of-life outcomes throughout study time points.

Health-related quality of life	*n*	SOC (mean ± DE)	*n*	EPC (mean ± DE)	Mean difference (95% CI)	*P*-value(*t* Student)
Global health status						
Baseline	67	48.5 ± 26.6	70	56.9 ± 25.4	8.3 (−0.4, 17.1)	0.061
Sixcycles	34	55.1 ± 23.7	42	56.9 ± 25.3	1.7 (−9.5, 13.1)	0.753
*P*		0.508		0.263		
ΔBaseline 6 cycles	34	3.1 ± 27.7	42	−4.9 ± 28.3	−8.1 (−21.0, 4.7)	0.213
Physical functioning						
Baseline	67	62.5 ± 28.1	70	64.5 ± 25.2	1.9 (−7.0, 11.0)	0.665
Six cycles	34	72.1 ± 24.2	42	65.0 ± 27.5	−7.0 (−19.0, 4.9)	0.245
*P*		0.536		0.429		
ΔBaseline— 6 cycles	34	2.3 ± 21.9	42	−3.6 ± 29.6	−6.0 (−18.1, 6.1)	0.329
Role functioning						
Baseline	67	54.7 ± 35.7	69	60.1 ± 32.7	5.4 (−6.2, 17.0)	0.358
Six cycles	34	65.1 ± 30.5	42	65.0 ± 30.9	−0.1 (−14.2, 14.0)	0.987
*P*		0.827		0.832		
ΔBaseline—6 cycles	32	−4.6 ± 24.0	38	1.7 ± 42.0	6.4 (−9.6, 22.5)	0.426
Emotional functioning						
Baseline	66	65.1 ± 26.9	70	66.6 ± 23.4	1.5 (−7.0, 10.0)	0.727
6 cycles	34	76.7 ± 24.4	42	73.8 ± 20.7	−2.9 (−13.2, 7.4)	0.576
*P*		0.215		0.582		
ΔBaseline—6 cycles	34	5.8 ± 27.1	42	1.9 ± 23.1	−3.8 (−15.3, 7.5)	0.501
Cognitive functioning						
Baseline	67	78.3 ± 28.2	70	78.3 ± 22.9	−0.02 (−8.7, 8.6)	0.996
Six cycles	34	78.4 ± 25.1	42	81.7 ± 25.7	3.3 (−8.3, 15.0)	0.574
*P*		0.391		0.938		
ΔBaseline—6 cycles	34	−3.9 ± 26.2	42	−0.3 ± 33.0	3.5 (−10.3, 17.4)	0.615
Social functioning						
Baseline	67	69.9 ± 33.4	70	65.4 ± 33.1	−4.4 (−15.6, 6.8)	0.438
Six cycles	34	65.1 ± 29.6	42	63.8 ± 31.4	−1.3 (−15.4, 12.7)	0.854
*P*		0.467		0.086		
ΔBaseline—6 cycles	34	−4.9 ± 38.8	42	−10.7 ± 39.4	−5.8 (−23.8, 12.1)	0.522
Fatigue						
Baseline	67	48.2 ± 26.8	70	44.9 ± 25.8	−3.3 (−12.2, 5.5)	0.460
Six cycles	34	38.5 ± 23.7	42	42.3 ± 25.2	3.7 (−7.5, 15.0)	0.509
*P*		0.407		0.897		
ΔBaseline—6 cycles	34	−4.2 ± 29.4	42	0.5 ± 26.4	4.7 (−8.0, 17.5)	0.459
Nausea and vomiting						
Baseline	66	25.0 ± 30.4	69	16.4 ± 18.4	−8.5 (−17.2, 0.05)	0.051
Six cycles	34	24.5 ± 31.3	42	16.2 ± 16.6	−8.2 (−19.4, 2.9)	0.146
*P*		0.698		0.719		
ΔBaseline—6 cycles	34	−2.4 ± 36.4	42	−1.1 ± 21.2	1.2 (−12.1, 14.6)	0.851
Pain						
Baseline	67	40.0 ± 30.9	70	53.5 ± 90.3	−0.7 (−10.8, 9.3)	0.881
Six cycles	34	34.8 ± 27.6	42	34.9 ± 28.4	0.1 (−12.7, 13.0)	0.986
*P*		0.160		0.092		
ΔBaseline—6 cycles	34	6.8 ± 27.8	42	-4.3 ± 31.6	−11.2 (−25.0, 2.5)	0.109
Dyspnea						
Baseline	65	31.2 ± 33.7	70	27.1 ± 26.7	−4.1 (−14.4, 6.2)	0.430
Six cycles	34	19.6 ± 26.1	42	21.4 ± 27.3	1.8 (−10.5, 14.1)	0.769
*P*		0.598		0.323		
ΔBaseline—6 cycles	33	−3.0 ± 32.6	42	−3.9 ± 25.7	−0.9 (−14.3, 12.4)	0.890
Appetite loss						
Baseline	66	39.8 ± 34.2	70	38.0 ± 32.2	−1.8 (−13.0, 9.4)	0.752
Six cycles	34	28.4 ± 30.8	42	35.7 ± 28.8	7.2 (−6.4, 20.9)	0.293
*P*		0.619		0.900		
ΔBaseline—6 cycles	34	−3.9 ± 45.5	42	0.7 ± 40.6	4.7 (−15.0, 24.4)	0.635
						

### Anxiety and Depression

At baseline, an assessment of the anxiety subscale of the HADS instrument showed that 44.1% of patients included in the EPC arm of the trial and 56.3% of patients allocated to the SOC arm had anxiety symptoms (*P* = .177). The rate of anxiety among patients in the experimental arm increased to 59.3%, 64.4%, and 74.6% at the 2nd, 4th, and 6th cycle evaluations, respectively. Meanwhile, patients in the SOC arm had an increased rate of anxiety earlier in the study, with 70.3% of patients in the 2nd cycle evaluation and reaching 75% and 74.6% by the 4th and 6th cycle evaluation. The differences between both study arms in anxiety were non-significant at all study points (Supplementary Fig. S3). Regarding depression, 42.4% and 45.3% of patients in the EPC and SOC arm had depression symptoms baseline, respectively. In the experimental arm, rate of depression increased to 54.2%, 69.5%, and 71.2% throughout the 2nd, 4th, and 6th cycle evaluation, respectively.

Furthermore, in the SOC arm, rate of depression at 2nd, 4th, and 6th cycle evaluations were 68.8%, 73.4%, and 82.8%. Nevertheless, the rate of depression by 6th cycle was numerically higher in the control group compared with the experimental group, this difference was not statistically significant (*P* = .124; Supplementary Fig. S4). Data regarding the scores of HADS anxiety and depression subscales are summarized in Supplementary Table 5.

### Caregivers Assessment

No differences between levels of burden were identified within the caregivers, except after 6 cycles when caregivers allocated to the EPC arm (median 23 points [*p*25.75: 13.0-34.3]) had a higher score vs. SOC arm (median 14 points [*p*25-75: 8.0-22.5], *P* = .016). No caregiver had values exceeding 47 points, as such, none was considered to be experiencing mild-to-severe burden.

## Discussion

Current oncology guidelines favor the implementation of EPC for patients with advanced neoplasms, highlighting that this model should be integrated into the comprehensive cancer care alongside active therapy.^21,41,42^ Although understanding the diverse mechanisms that might be responsible for the improvements achieved through EPC is a research priority, EPC is consistently strongly recommended due to benefits outweighing harms, with an intermediate level of evidence quality.^21^ The most recent recommendations are supported in the latest myriad of studies, including several high-quality randomized clinical trials, which have addressed the many benefits of offering palliative care early in the course of the disease, including improvements in QoL, mood, symptom control, and—much less understood—improved overall survival.^10,22-24,26,43-46^

To the best of our knowledge, our study represents the first randomized clinical trial to evaluate the effect of EPC in patients with metastatic NSCLC in Latin America, overcoming one of the most cited limitations in the aforementioned studies, conducted mainly in white populations, with scarce racial and ethnic representation and thus hindering the generalization of the results.^10,24,44^ This is particularly important when considering the global scenario in terms of cancer epidemiology and the availability of palliative care services.^47^ In this regard, data from global repositories estimates that two-thirds of cancer deaths worldwide occur among individuals in LMIC.^47^ In this specific setting, evidence regarding the benefits of EPC is much lacking, and so patients from underprivileged areas do not have access to standard practices elsewhere.^47^ In the second edition of the Global Atlas of Palliative Care, the results showed a considerable increase in palliative care services from 2014 (16 000 services caring for 3 million patients) to 2020 (25 000 services caring for 7 million patients).^48^ Though this may seem encouraging, results also highlight that worldwide 56.8 million people require palliative care, thus, currently meeting the needs of merely 12% of individuals.^47,48^ Moreover, 76% of the adults who require palliative care live in LMIC, among which only 10%-38% provide palliative care as primary healthcare, compared with 81% of high-income countries.^48^ The data stresses the paradox of the highest needing area having the lowest current access and must incite a surge of efforts to provide access to all in need of this international human right.^48^ The development of high-quality regional research to highlight the benefits of this intervention among local populations and adhering to the context of this setting can drive policy implementation to benefit patients.^49^

Among the barriers we faced to conduct this study were the lack of resources from the hospital itself and from the patients. Some of them did not have the means to attend their appointments, being that, because their primary caregiver could not afford to take a day off from work. In addition, many of the patients were from different cities which made the treatment a lot harder. Another barrier was the misconception about palliative care among patients, as sometimes they believed it was not a real treatment or it meant the end of life.

Results from our study certainly encourage the implementation of EPC among patients with advanced NSCLC in Latin America. Among the key results from the primary outcome of our trial, we demonstrated a significantly prolonged OS among patients allocated to the experimental arm of the study (18.1 vs 10.5 months); this benefit was independent of other variables, as stressed by the multivariate analysis. In addition, we highlight that this benefit is even more pronounced among patients with a good performance status (0–1) at the time of EPC referral (21.1 vs 13.8 months) and with a good global QoL (38.7 vs 21.4 months). These results closely resemble previous studies that have evaluated the role of EPC among patients with advanced cancer; in a pivotal study by Temel et al, the authors identified a significant improvement in survival among patients with metastatic NSCLC allocated to EPC, compared with those receiving SOC (11.6 vs 8.9 months; *P* = .02).^10^ Our findings are consistent with Temel’s study validating the role of EPC in this particular population in a LMIC. Another study that evaluated the early vs. delayed initiation of concurrent palliative oncology care also identified an improved OS among patients in the early arm (18.3 vs. 8.9 months). It is important to emphasize that both trials were designed and implemented in the last decade, so a critical finding also pertains to the fact that EPC continues to fare well in terms of survival in the present clinical context^26^ The exact mechanism whereby EPC may improve survival is yet to be defined.^24,50^ Potential mechanisms include the prognostic effect of an initial good performance status and a good quality of life as observed in this study along with an intensive care in terms of palliative care besides usual treatment. Given the diversity of treatment today, it would be worth evaluating it by homogenizing the treatment received by patients to isolate the factors that promote better OS associated with EPC.

Interestingly, despite having a favorable outcome in survival in our study, patients allocated to the EPC arm of the trial did not have a significant improvement in QoL throughout the study evaluations. Previously, a trial by Bakitas et al ^24^ reported similar findings, emphasizing that early implementation of palliative care remarkably improved survival. Still, the authors did not detect differences in QoL, symptom impact, and patient mood.^24^ This study, entitled the ENABLE trial, highlights that palliative care is usually offered late among patients with advanced neoplasms, which hampers the benefit reaped by patients.^24^ In this case, unlike in previous trials,^10,23^ the benefit is restricted to survival data, lacking significance in other patient-reported outcomes. This lack of significance could be attributed to diverse reasons, including those reported by Bakitas, which include measurement insensitivity, a ceiling effect, or a stabilizing effect from the intervention.^24^ Similar to their trial, patients allocated to the SOC arm in our study could be freely referred to PC at any time, either at their request or by the submission of their clinician.^51^ In this sense, it could be that as more information became available in the last decade regarding the benefits of PC for patients with cancer, clinicians were more aware of the need for referral. In fact, most palliative care studies stress that the intervention’s true effect might be diluted due to this design, in which no patient is denied access to palliative care services throughout the study.^10,24^ In a subsequent editorial, Gomez discussed the many advantages of offering palliative care earlier rather than later, noting that “even with evidence of no effect on other outcomes, survival improvement may be enough to justify the introduction of palliative care.” Results from our study are in complete agreement with this relevant statement.^52^

Though EPC is consistently viewed as a cornerstone part of the continuum of care for patients with cancer, the specific areas that translate into an improved outcome are amiss. In a recent study by Borelli et al,^43^ the authors dissect the changes in patients’ with cancer and caregivers’ disease perceptions and analyze these findings. Through a series of elegant semi-structured interviews, the authors conclude that EPC allows a prompt resolution of symptoms and their consequences; further, the intervention provides a sense of empowerment and a person-focused approach among this patient group. Overall, participants enrolled, as well as their caregivers, perceive EPC to be beneficial. Audiotapes from the interviews show that after receiving EPC, patients improved their ability to cope and acceptance skills, which may translate into benefits in the perception of the disease, expectations for end-of-life, less depression, and therefore improved outcomes.^43^

Regarding the psychological variables, such as anxiety and depression, which increased during the study, this has been seen before in other studies. We know that depression can be more significant with the number of cycles and anxiety follows a similar trend impacting on QoL. Of note, none of the caregivers in our study exceeded the score of 47 points considered as mild-to-severe burden but did have a higher score after 6 cycles if they were in the EPC arm, which could be related to the need to attend to a greater number of appointments compared to the SoC arm.

Overall, our data provide evidence to support the idea that even in resource-limited settings,^53-55^ implementation of EPC is possible among patients with metastatic NSCLC cancer,^56^ and despite the challenges of offering patients in LMICs access to costly novel therapeutic approaches,^57^ palliative care services may offer significant survival benefits as previously reported by other prospective study^10^ while not incurring in overwhelming expense and remaining cost-effective.^58^ Other advantages include the systematic implementation of care of the patient linking together the treatable approach of the disease with the palliative. We have to overcome multiple barriers including misconceptions of the role of palliative care and fear of to use opioids and other agents as part of symptom management, cultural and social barriers about death and the process of dying, education of patients but also of health professionals about the relevance of working with a multidisciplinary team since the diagnosis and not later in the course of the disease. Therefore, EPC must be implemented as SOC in LMIC for all patients with metastatic NSCLC who agree to receive this service.

Several limitations of the study deserve mention. It was performed at a single, tertiary cancer center with a focused group in thoracic oncology and palliative care, thus impeding an extended replication of the findings in a variety of cancer populations. Although we did not detect differences in baseline characteristics, discrepancies in unmeasured characteristics could limit applicability. Allowing access to palliative care in the control arm may be biased toward the null hypothesis possibly having an impact on the survival outcome. However, less than 35% of the control group receive palliative care in a median of 6.2 (95% CI, 3.6-23.1 months). Moreover, it is possible that the benefit in survival occurred from unmeasured palliative care effects. For instance, EPC provides anticipatory guidance about advance care planning and goals of care, which may affect OS. Although chemotherapy use was similar between both groups in our study, there were more patients in the control arm who did not receive chemotherapy, which certainly influenced a worse OS. The lack of availability to perform a mutational profile on all the patients is one of the study’s limitations, so it is not possible to assess their real impact affecting the survival of the patients and that could have diluted the intervention impact. Both parts of the study (clinicians and patients) were not masked. We allowed patients in the control arm could be referred to palliative care as needed, and all the results obtained from this group were analyzed in combination with their assigned group which may also have diluted our findings. Considering as timepoints the number of cycles of treatment could be a limitation as this could be variable between patients. Lastly, a baseline survey assessment was given after patients were randomized which might influence how patients respond to their baseline questionnaires, however, no differences in GQoL were found between groups. We acknowledge that the latter could be a major limitation of the current study but does not preclude from taking the present results as a remarkable contribution as this study shows that EPC is highly important and provides benefits to patients with metastatic NSLSC from a resource limited-setting.

## Conclusion

In this randomized clinical trial, patients with metastatic NSCLC receiving EPC had a significantly prolonged OS compared with those receiving SOC alone in a LMIC setting showing that is a feasible and clinically meaningful strategy. Interestingly, the benefit in terms of survival is more pronounced among the subgroup of patients who have a good performance status and good global QoL at diagnosis.

## Supplementary Material

Supplementary material is available at *The Oncologist* online.

oyae050_suppl_Supplementary_Figures_S1-S4

oyae050_suppl_Supplementary_Tables_1-5

## Data Availability

The data underlying this article are available in the article and in its online supplementary material.

## Previous Presentation

An interim analysis with partial information of allocated patients was presented during the World Conference on Lung Cancer, Yokohama, Japan, 2017.
