# Supplementary material for: Early Incorporation to Palliative Care (EPC) in Patients With Advanced Non-Small Cell Lung Cancer: The PACO Randomized Clinical Trial
Source: Oncologist. 2024 Apr 1;29(10):e1373–85. doi: 10.1093/oncolo/oyae050 (PMC11449095; doi:10.1093/oncolo/oyae050)
Supplement: oyae050_suppl_Supplementary_Figures_S1-S4 [file oyae050_suppl_supplementary_figures_s1-s4.docx]

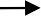
**Supplementary Figure S1.** Study design and procedures

Enrollment

Assessed for eligibility (n=201)

Baseline evaluation (n=146)

Randomized (n=146)

Allocated to receive early- palliative care (n=73)

Allocation

Allocated to receive Standard of care (n=73)

Begin treatment

Evaluation of:

- Symptoms
- Nutritional status
- Psychological status

Evaluation of:

- Symptoms (ESAS)
- Nutritional status
- Psychological status (HADs, QoL)
- Social (Zarit)

**The patient was assessed at the end of every 2 cycles, until death or loss of follow-up**

**Supplementary Figure S2.** Overall survival between groups according to global health of quality of life.


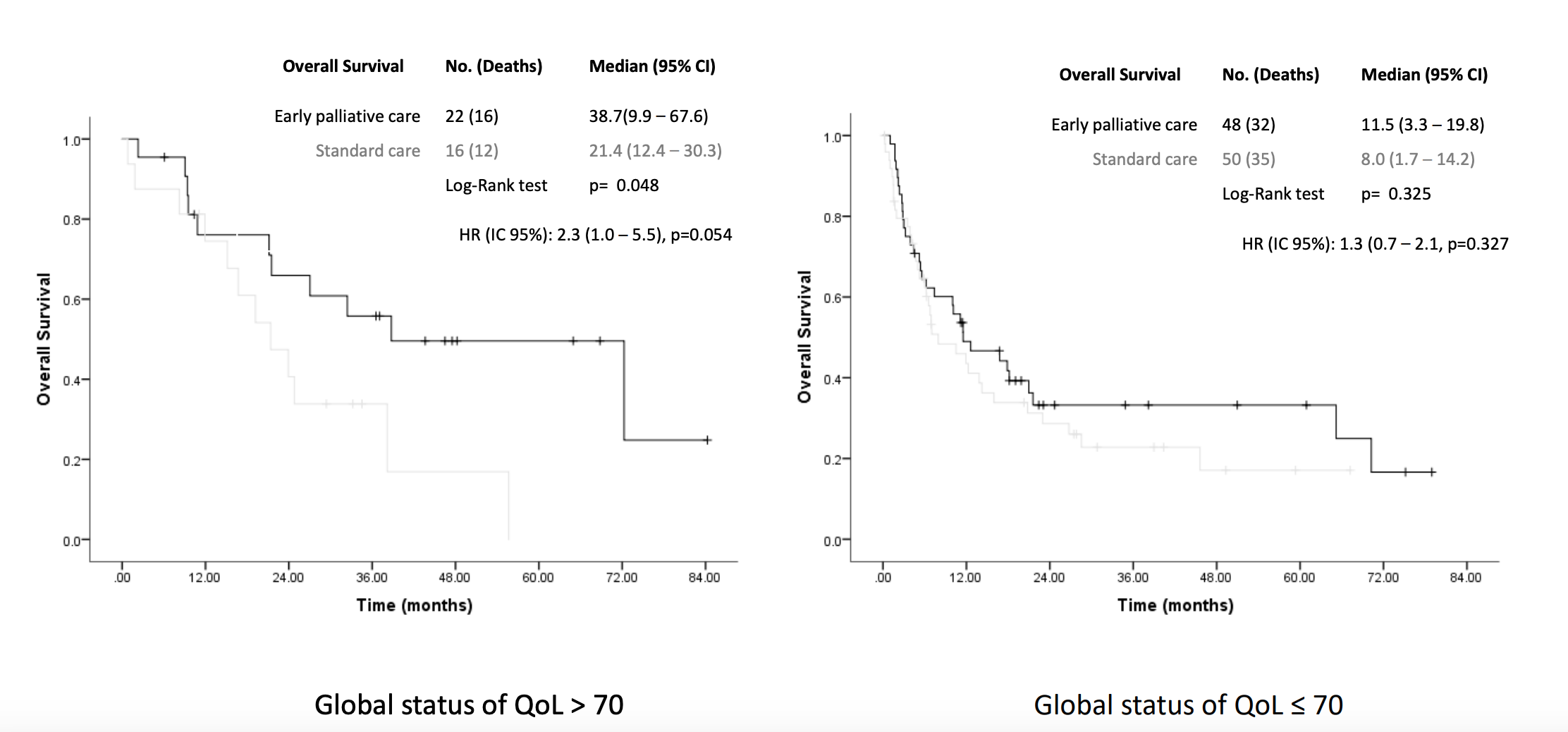


**Supplementary Figure S3**. Percentage of patients with anxiety throughout the study.

p= 0.078

p= 0.017

p= 0.029


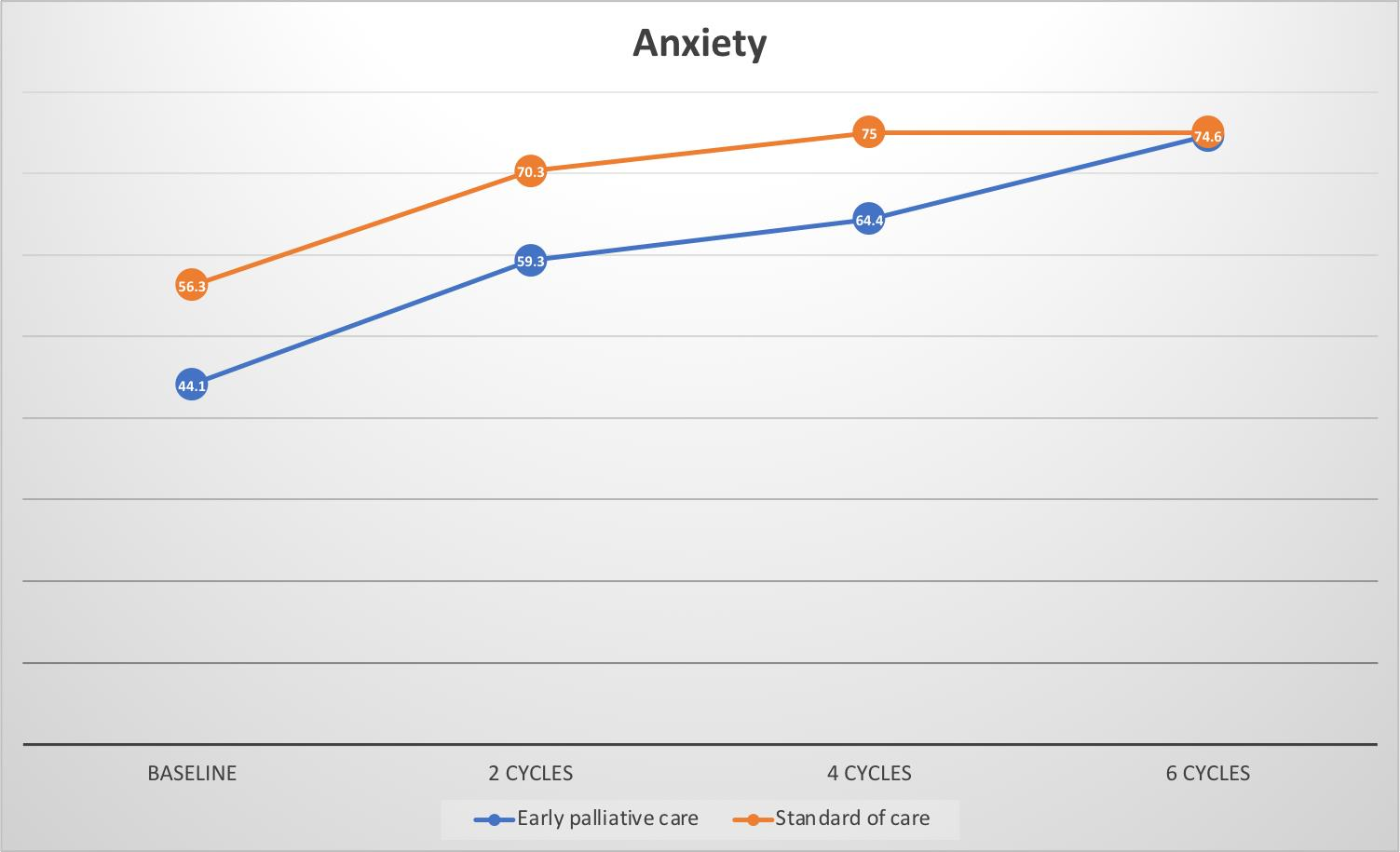


p= 0.078

p= 0.023

p <0.001

**Supplementary Figure S4**. Percentage of patients with depression throughout the study.

p= 0.004

p= 0.001

p= <0.001


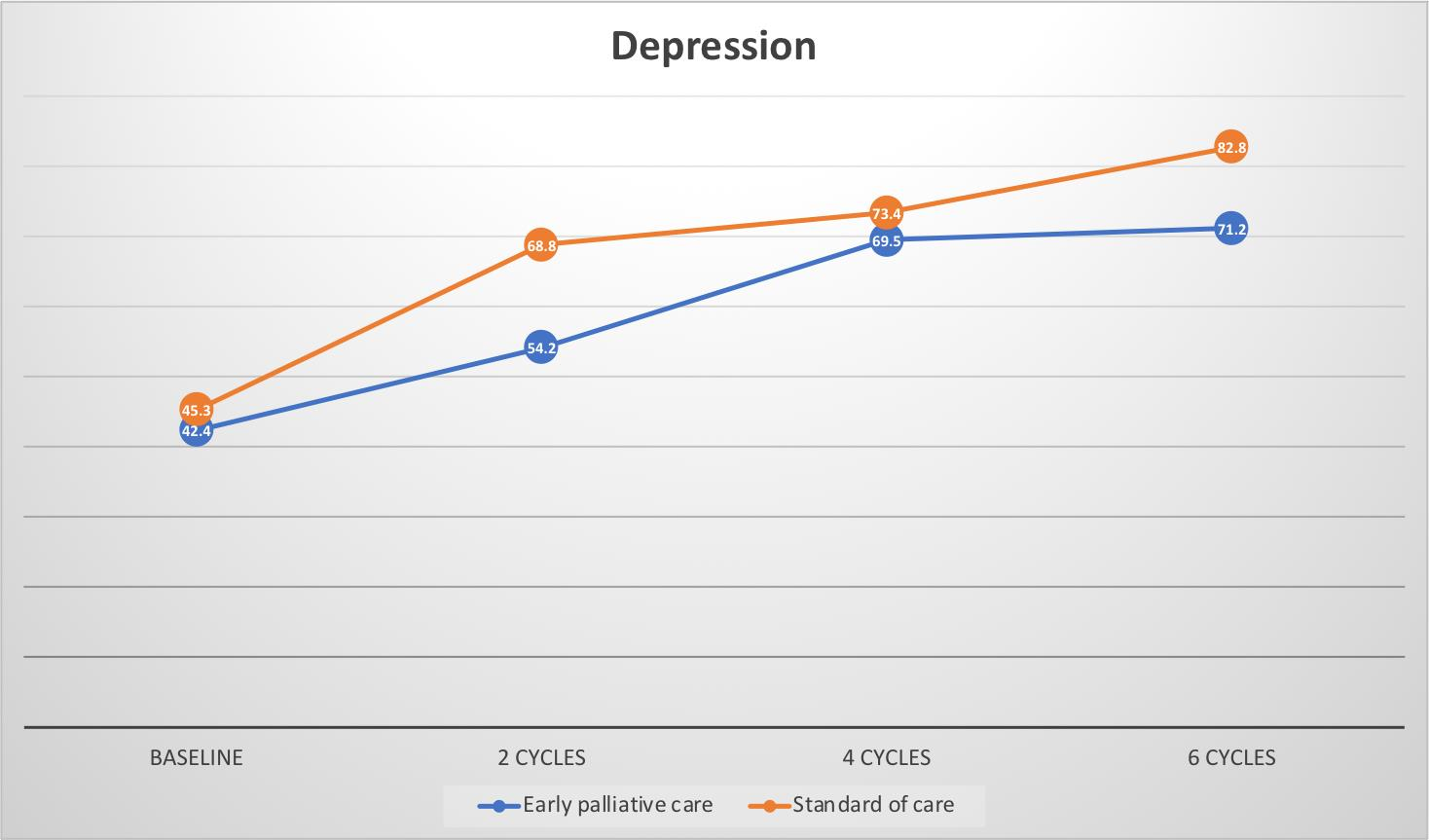


p= <0.001

p= 0.002

p= 0.143
