# Supplementary material for: Early Incorporation to Palliative Care (EPC) in Patients With Advanced Non-Small Cell Lung Cancer: The PACO Randomized Clinical Trial
Source: Oncologist. 2024 Apr 1;29(10):e1373–85. doi: 10.1093/oncolo/oyae050 (PMC11449095; doi:10.1093/oncolo/oyae050)
Supplement: oyae050_suppl_Supplementary_Tables_1-5 [file oyae050_suppl_supplementary_tables_1-5.pdf]

Supplementary Table 1. Health-related quality of life among patients with a good performance status (ECOG 0-1)

Health related quality of life	**n**	**C** (mean ± DE)	**n**	**EPC** (mean ± DE)	**Mean difference (95% CI)**	p (t student)
Global health status						
Baseline	53	46.6 ± 26.3	59	59.0 ± 24.4	12.3 (2.8 – 21.8)	**0.011**
2 cycles	33	60.3 ± 22.1	45	60.9 ± 26.6		
4 cycles	32	54.9 ± 23.9	41	63.0 ± 25.4		
6 cycles	28	54.7 ± 25.2	37	57.4 ± 25.3	2.6 (-10.0 – 15.3)	0.675
P Baseline - 6 cycles		0.405		0.124		
Δ Baseline - 6 cycles	28	4.4 ± 27.9	37	-6.9 ± 26.9	-11.4 (-25.1 – 2.2)	0.100
Physical functioning						
Baseline	53	68.0 ± 24.6	59	66.6 ± 23.4	-1.3 (-10.4 – 7.6)	0.762
2 cycles	35	73.3 ± 21.3	48	69.8 ± 20.6		
4 cycles	32	71.4 ± 24.3	41	68.1 ± 25.2		
6 cycles	28	75.2 ± 20.8	37	54.7 ± 26.3	-8.2 (-20.2 -3.8)	0.179
P Baseline - 6 cycles		0.956		0.496		
Δ Baseline - 6 cycles	28	0.2 ± 22.4	37	-3.0 ± 27.1	-3.2 (-15.9 – 9.3)	0.603
Role functioning						
Baseline	53	60.3 ± 33.6	58	61.2 ± 32.5	0.8 (-11.6 – 13.2)	0.895
2 cycles	35	68.0 ± 29.8	48	64.2 ± 30.5		
4 cycles	32	63.0 ± 33.7	41	64.6 ± 34.1		
6 cycles	28	66.0 ± 29.5	37	66.2 ± 30.0	0.1 (-14.7 – 15.0)	0.984
P Baseline - 6 cycles		0.565		0.877		

Δ Baseline - 6 cycles	26	-6.4 ± 25.8	34	-1.9 ± 41.5	4.4 (-13.0 – 21.9)	0.613
Emotional functioning						
Baseline	53	69.1 ± 23.4	59	68.3 ± 22.2	-0.8 (-9.3 – 7.7)	0.850
2 cycles	33	76.0 ± 19.7	45	73.7 ± 23.2		
4 cycles	32	74.7 ± 24.2	41	72.1 ± 27.2		
6 cycles	28	78.5 ± 20.5	37	75.6 ± 19.1	-2.8 (-12.8 – 7.0)	0.561
P Baseline - 6 cycles		0.260		0.337		
Δ Baseline - 6 cycles	28	6.2 ± 28.7	37	3.3 ± 21.1	-2.8 (-15.2 – 9.4)	0.644
Cognitive functioning						
Baseline	53	81.4 ± 25.0	59	79.9 ± 21.8	-1.5 (-10.2 – 7.2)	0.735
2 cycles	33	82.3 ± 19.0	45	81.1 ± 23.1		
4 cycles	32	81.2 ± 19.2	40	77.4 ± 24.6		
6 cycles	28	76.7 ± 24.9	37	83.7 ± 22.7	6.9 (-4.8 – 18.8)	0.243
P Baseline - 6 cycles		0.141		0.924		
Δ Baseline - 6 cycles	28	-7.7 ± 27.0	37	0.4 ± 28.7	8.1 (-5.8 – 22.2)	0.248
Social functioning						
Baseline	53	78.3 ± 27.4	59	65.5 ± 32.4	-12.7 (-24.0 – (-1.4))	**0.027**
2 cycles	33	64.6 ± 26.9	45	69.2 ± 27.7		
4 cycles	32	66.1 ± 26.9	41	63.0 ± 28.9		
6 cycles	28	68.4 ± 26.1	37	62.6 ± 31.7	-5.8 (-20.6 – 8.9)	0.432
P Baseline - 6 cycles		0.330		0.066		
Δ Baseline - 6 cycles	28	-7.1 ± 38.0	37	-12.1 ± 39.0	-5.01 (-24.3 – 14.3)	0.606
Fatigue						
Baseline	53	44.6 ± 26.1	59	44.0 ± 26.0	-0.5 (-10.3 – 9.2)	0.906

2 cycles	35	39.0 ± 24.6	48	40.9 ± 26.1		
4 cycles	32	40.9 ± 24.0	41	42.2 ± 26.6		
6 cycles	28	37.6 ± 23.6	37	41.7 ± 24.9	4.0 (-8.1 – 16.2)	0.510
P Baseline - 6 cycles		0.695		0.721		
Δ Baseline - 6 cycles	28	-2.3 ± 31.7	37	1.5 ± 25.4	3.8 (-10.2 – 18.0)	0.586
Nausea and vomiting						
Baseline	53	24.2 ± 29.1	58	15.2 ± 17.4	-8.9 (-18.1 – 0.1)	0.055
2 cycles	35	26.6 ± 27.4	48	21.8 ± 25.3		
4 cycles	31	18.2	41	23.5 ± 28.6		
6 cycles	28	19.6 ± 27.2	37	15.3 ± 15.8	-4.3 (-15.0 – 6.4)	0.425
P Baseline - 6 cycles		0.170		0.790		
Δ Baseline - 6 cycles	28	-8.9 ± 33.4	37	-0.9 ± 20.3	8.0 (-6.4 – 22.4)	0.236
Pain						
Baseline	53	35.2 ± 29.1	59	38.7 ± 27.9	3.4 (-7.2 – 14.1)	0.520
2 cycles	35	30.4 ± 24.4	46	33.6 ± 26.6		
4 cycles	32	31.7 ± 22.5	41	38.2 ± 34.0		
6 cycles	28	35.7 ± 26.3	37	35.5 ± 28.9	-0.1 (-14.0 – 13.8)	0.985
P Baseline - 6 cycles		**0.039**		0.115		
Δ Baseline - 6 cycles	28	11.3 ± 27.6	37	-29.7 ± 112.0	-41.0 (-84.4 – 2.3)	0.063
Dyspnea						
Baseline	52	29.4 ± 30.7	59	25.9 ± 27.0	-3.4 (-14.3 – 7.3)	0.525
2 cycles	35	20.9 ± 24.3	48	27.0 ± 31.2		
4 cycles	32	23.9 ± 21.1	41	25.2 ± 30.5		
6 cycles	28	19.0 ± 23.0	37	19.8 ± 25.4	0.7 (-11.4 – 12.9)	0.900
P Baseline - 6 cycles		0.587		0.281		
Δ Baseline - 6 cycles	27	-3.7 ± 35.0	37	-4.5 ± 25.0	-0.8 (-15.7 – 14.1)	0.915

Insomnia						
Baseline	53	40.2 ± 35.4	59	37.8 ± 29.9	-2.3 (-14.6 – 9.8)	0.699
2 cycles	35	30.4 ± 28.4	47	25.5 ± 25.2		
4 cycles	32	33.3 ± 31.6	41	22.7 ± 28.3		
6 cycles	28	30.9 ± 29.9	37	26.1 ± 28.4	-4.8 (-19.4 – 9.7)	0.511
P Baseline - 6 cycles		0.892		0.078		
Δ Baseline - 6 cycles	28	-1.1 ± 45.7	37	-9.9 ± 33.2	-8.7 (-28.2 – 10.8)	0.377
Appetite loss						
Baseline	53	38.3 ± 34.2	59	36.1 ± 31.7	-2.2 (-14.5 – 10.1)	0.724
2 cycles	35	27.6 ± 33.8	48	38.1 ± 35.0		
4 cycles	32	35.4 ± 33.8	41	35.7 ± 34.4		
6 cycles	28	26.1 ± 30.5	37	32.4 ± 26.6	6.2 (-7.9 – 20.4)	0.383
P Baseline - 6 cycles		0.449		0.674		
Δ Baseline - 6 cycles	28	-7.1 ± 49.1	37	-2.7 ± 38.7	4.4 (-17.3 – 26.2)	0.685
Constipation						
Baseline	53	20.7 ± 28.6	59	27.6 ± 30.4	6.9 (-4.1 – 18.0)	0.219
2 cycles	35	21.9 ± 27.9	48	19.4 ± 29.0		
4 cycles	32	18.7 ± 28.0	41	21.9 ± 25.3		
6 cycles	28	17.8 ± 21.2	37	24.3 ± 26.8	6.4 (-5.8 – 18.7)	0.298
P Baseline - 6 cycles		0.599		0.544		
Δ Baseline - 6 cycles	28	-3.5 ± 35.5	37	-3.6 ± 35.8	-0.03 (-17.8 – 17.8)	0.997
Diarrhea						
Baseline	53	8.8 ± 19.7	59	12.9 ± 21.4	4.1 (-3.5 – 11.9)	0.287
2 cycles	34	16.6 ± 26.2	45	17.0 ± 23.1		
4 cycles	32	10.4 ± 15.6	41	10.5 ± 21.6		

6 cycles	28	14.2 ± 24.7	37	13.5 ± 16.5	-0.7 (-11.0 – 9.4)	0.881
P Baseline - 6 cycles		0.861		0.661		
Δ Baseline - 6 cycles	28	1.1 ± 35.6	37	1.8 ± 24.7	0.6 (-14.3 – 15.6)	0.935
Financial difficulties						
Baseline	53	51.5 ± 31.7	59	61.5 ± 34.3	10.0 (-2.4 – 22.4)	0.113
2 cycles	33	66.6 ± 28.8	45	57.0 ± 34.5		
4 cycles	31	62.3 ± 29.4	41	63.4 ± 30.5		
6 cycles	28	63.0 ± 29.1	37	65.7 ± 38.0	2.6 (-13.9 – 19.3)	0.750
P Baseline - 6 cycles		0.164		0.680		
Δ Baseline - 6 cycles	28	10.7 ± 39.6	37	2.7 ± 39.5	-8.0 (-27.8 – 11.8)	0.422
Dyspnea 2						
Baseline	53	29.3 ± 23.4	58	28.1 ± 25.6	-1.1 (-10.4 – 8.0)	0.800
2 cycles	35	27.9 ± 24.8	48	26.6 ± 25.4		
4 cycles	32	24.6 ± 22.5	40	24.1 ± 24.8		
6 cycles	28	22.6 ± 18.2	37	19.2 ± 23.3	-3.3 (-14.0 – 7.2)	0.527
P Baseline - 6 cycles		0.854		0.343		
Δ Baseline - 6 cycles	28	-0.7 ± 22.6	37	-4.2 ± 26.6	-3.4 (-15.9 – 9.0)	0.588
**Coughing**						
Baseline	53	53.4 ± 27.2	58	37.9 ± 30.8	-15.5 (-26.5 – (-4.5))	**0.006**
2 cycles	35	39.0 ± 24.9	48	31.2 ± 28.6		
4 cycles	32	34.3 ± 19.8	40	29.9 ± 23.6		
6 cycles	28	29.7 ± 22.8	37	20.7 ± 22.7	-9.0 (-20.4 – 2.3)	0.118
P Baseline - 6 cycles		**0.032**		**0.034**		
Δ Baseline - 6 cycles	28	-13.0 ± 30.5	37	-13.5 ± 37.2	-0.4 (-17.7 – 16.8)	0.962
Haemoptysis						

Baseline	53	17.6 ± 25.8	58	6.8 ± 16.2	-10.7 (-18.7 – (-2.6))	**0.009**
2 cycles	35	4.7 ± 11.8	48	3.4 ± 12.3		
4 cycles	32	6.2 ± 13.2	40	0.8 ± 5.2		
6 cycles	28	7.1 ± 22.8	37	5.4 ± 12.4	-1.7 (-10.5 – 7.1)	0.696
P Baseline - 6 cycles		0.165		0.324		
Δ Baseline - 6 cycles	28	-8.3 ± 30.9	37	-2.7 ± 16.4	5.6 (-7.4 – 18.6)	0.387
Sore mouth						
Baseline	53	3.7 ± 12.5	58	9.1 ± 20.5	5.4 (-0.9 – 11.7)	0.093
2 cycles	35	8.5 ± 18.6	48	9.0 ± 20.3		
4 cycles	32	7.2 ± 16.3	40	11.6 ± 25.6		
6 cycles	28	5.9 ± 15.8	36	6.4 ± 15.5	0.5 (-7.3 – 8.4)	0.894
P Baseline - 6 cycles		0.999		0.413		
Δ Baseline - 6 cycles	28	0 ± 15.7	36	-2.7 ± 20.1	-2.7 (-12.0 – 6.4)	0.550
Dysphagia						
Baseline	53	20.7 ± 30.8	58	10.9 ± 20.1	-9.8 (-19.7 – 0.08)	0.052
2 cycles	35	19.0 ± 30.5	48	20.8 ± 30.4		
4 cycles	32	14.5 ± 25.3	40	15.0 ± 29.1		
6 cycles	28	4.7 ± 11.8	37	14.4 ± 21.5	9.6 (1.2 – 18.0)	**0.025**
P Baseline - 6 cycles		**0.013**		0.556		
Δ Baseline - 6 cycles	28	-13.0	37	2.7 ± 27.6	15.7 (2.2 – 29.3)	**0.023**
Peripheral neuropathy						
Baseline	53	23.2 ± 29.6	58	24.1 ± 52.9	0.8 (-15.4 – 17.2)	0.916
2 cycles	35	36.1 ± 31.6	48	24.9 ± 28.7		
4 cycles	32	35.4 ± 32.7	40	30.8 ± 30.5		

6 cycles	28	36.9 ± 29.1	37	32.4 ± 31.9	-4.4 (-19.8 – 10-.9)	0.564
P Baseline - 6 cycles		0.161		**0.022**		
Δ Baseline - 6 cycles	28	9.5 ± 34.9	37	15.3 ± 38.9	5.7 (-12.8 – 24.4)	0.538
Alopecia						
Baseline	53	22.6 ± 37.9	58	22.9 ± 33.7	0.3 (-13.1 – 13.8)	0.959
2 cycles	35	65.7 ± 37.4	48	55.5 ± 43.6		
4 cycles	32	64.5 ± 35.8	40	50.0 ± 44.6		
6 cycles	28	54.7 ± 43.7	36	36.1 ± 42.4	-18.6 (-40.3 – 3.0)	0.090
P Baseline - 6 cycles		**0.013**		**0.042**		
Δ Baseline - 6 cycles	28	28.5 ± 57.1	36	16.6 ± 47.4	-11.9 (-38.0 – 14.2)	0.366
Pain in chest						
Baseline	53	30.8 ± 31.2	58	24.1 ± 33.5	-6.6 (-18.8 – 5.5)	0.281
2 cycles	35	19.0 ± 23.2	48	24.9 ± 31.1		
4 cycles	32	18.7 ± 20.6	39	23.9 ± 29.5		
6 cycles	28	15.4 ± 26.4	37	22.5 ± 27.2	7.0 (-6.4 – 20.5)	0.300
P Baseline - 6 cycles		0.147		0.638		
Δ Baseline - 6 cycles	28	-9.5 ± 33.7	37	2.7 ± 34.5	12.2 (-4.9 – 29.3)	0.159
Pain in arm or shoulder						
Baseline	52	23.7 ± 31.1	58	27.0 ± 30.8	3.2 (-8.4 – 15.0)	0.579
2 cycles	35	18.0 ± 26.0	48	15.2 ± 25.6		
4 cycles	32	19.7 ± 25.2	40	14.9 ± 27.1		
6 cycles	27	30.8 ± 26.0	37	25.2 ± 29.8	-5.6 (-19.9 – 8.6)	0.434
P Baseline - 6 cycles		**0.019**		0.999		
Δ Baseline - 6 cycles	27	13.5 ± 28.1	37	0 ± 36.8	-13.5 (-30.5 – 3.3)	0.114

Pain in other parts						
Baseline	53	23.8 ± 23.9	57	27.0 ± 30.8	8.2 (-1.4 – 18.0)	0.096
2 cycles	34	25.4 ± 23.2	46	26.8 ± 19.3		
4 cycles	32	23.9 ± 22.7	38	25.4 ± 18.0		
6 cycles	27	27.1 ± 24.5	35	28.5 ± 25.7	1.4 (-11.5 – 14.3)	0.828
P Baseline - 6 cycles		0.542		0.762		
Δ Baseline - 6 cycles	27	3.7 ± 31.1	35	-1.9 ± 36.9	-5.6 (-23.3 – 12.1)	0.529

Supplementary Table 2. Health-related quality of life among patients with a poor performance status (ECOG >1)

Health related quality of life	n	**C** (mean ± DE)	n	**EPC** (mean ± DE)	**Mean difference (95% CI)**	p (t student)
Global health status						
Baseline	14	55.3 ± 27.6	11	45.4 ± 28.9)	-9.9 (-33.4 – 13.6)	0.393
2 cycles	9	56.4 ± 13.0	8	63.5 ± 20.3		
4 cycles	7	54.7 ± 28.8	8	51.0 ± 15.7		
6 cycles	6	56.9 ± 16.1	5	53.3 ± 28.0	-3.6 (-34.0 – 26.8)	0.794
P Baseline - 6 cycles		0.822		0.578		
Δ Baseline - 6 cycles	6	-2.7 ± 28.7	5	9.9 ± 36.9	12.7 (-31.9 – 57.4)	0.534
Physical functioning						
Baseline	14	41.9 ± 31.8	11	53.3 ± 32.2	11.4 (-15.2 – 38.1)	0.385
2 cycles	9	50.3 ± 29.2	8	54.9 ± 21.8		
4 cycles	7	52.3 ± 31.1	8	50.0 ± 13.8		
6 cycles	6	57.7 ± 35.1	5	50.6 ± 35.4	-7.1 (-55.4 – 41.2)	0.747

P Baseline - 6 cycles		0.150		0.730		
Δ Baseline - 6 cycles	6	12.2 ± 17.5	5	-8.0 ± 48.3	-20.2 (-67.9 – 27.4)	0.363
Role functioning						
Baseline	14	33.3 ± 36.3	11	54.5 ± 35.0	21.2 (-8.6 – 51.0)	0.155
2 cycles	9	48.1 ± 37.6	8	29.1 ± 31.8		
4 cycles	7	52.3 ± 41.3	8	58.3 ± 37.7		
6 cycles	6	61.1 ± 37.5	5	56.6 ± 40.1	-4.4 (-57.4 – 48.5)	0.854
P Baseline - 6 cycles		0.117		0.883		
Δ Baseline - 6 cycles	6	2.7 ± 12.5	4	33.3 ± 36	30.5 (-5.4 – 66.5)	0.086
Emotional functioning						
Baseline	13	48.7 ± 34.3	11	57.5 ± 28.7	8.8 (-18.2 – 35.9)	0.505
2 cycles	9	59.2 ± 33.4	8	80.2 ± 20.3		
4 cycles	7	76.1 ± 15.5	8	82.2 ± 10.3		
6 cycles	6	68.0 ± 39.2	5	60.0 ± 28.5	-8.0 (-55.8 – 39.7)	0.712
P Baseline - 6 cycles		0.624		0.639		
Δ Baseline - 6 cycles	6	4.1 ± 19.5	5	-8.3 ± 36.7	-12.4 (-51.5 – 26.5)	0.488
Cognitive functioning						
Baseline	14	66.6 ± 36.9	11	69.6 ± 27.7	3.0 (-24.6 – 30.7)	0.823
2 cycles	9	62.9 ± 32.0	8	70.8 ± 26.3		
4 cycles	7	80.9 ± 17.8	8	72.9 ± 21.7		
6 cycles	6	86.1 ± 26.7	5	66.6 ± 42.4	-19.4 (-66.8 – 27.9)	0.378
P Baseline - 6 cycles		**0.042**		0.818		

Δ Baseline - 6 cycles	6	13.8 ± 12.5	5	-6.6 ± 60.7	-20.5 (-95.3 – 54.2)	0.496
Social functioning						
Baseline	14	38.0 ± 36.0	11	65.1 ± 38.3	27.0 (-3.8 – 57.9)	0.083
2 cycles	9	37.0 ± 27.3	8	74.9 ± 34.5		
4 cycles	7	57.1 ± 38.3	8	64.5 ± 35.0		
6 cycles	6	50.0 ± 42.1	5	73.3 ± 30.2	23.3 (-27.8 – 74.4)	0.329
P Baseline - 6 cycles		0.771		0.999		
Δ Baseline - 6 cycles	6	5.5 ± 44.3	5	0.0 ± 45.6	-5.5 (-67.7 – 56.6)	0.843
Fatigue						
Baseline	14	61.9 ± 26.0	11	49.4 ± 25.0	-12.4 (-33.7 – 8.9)	0.241
2 cycles	9	55.5 ± 28.3	8	58.3 ± 16.5		
4 cycles	7	41.2 ± 25.4	8	50.0 ± 14.5		
6 cycles	6	42.5 ± 25.7	5	46.6 ± 30.8	4.0 (-34.4 – 42.5)	0.816
P Baseline - 6 cycles		0.058		0.697		
Δ Baseline - 6 cycles	6	-12.9 ± 12.9	5	-6.6 ± 35.6	6.2 (-28.8 – 41.4)	0.695
Nausea and vomiting						
Baseline	13	28.2 ± 36.2	11	22.7 ± 22.6	-5.4 (-31.6 – 20.7)	0.669
2 cycles	9	42.5 ± 25.1	8	29.1 ± 26.3		
4 cycles	7	33.3 ± 25.4	8	33.3 ± 26.7		
6 cycles	6	47.2 ± 41.3	5	23.3 ± 22.3	-23.8 (-70.8 – 23.0)	0.279
P Baseline - 6 cycles		0.129		0.815		
Δ Baseline - 6 cycles	6	27.7 ± 37.5	5	-3.3 ± 29.8	-31.1 (-78.1 – 15.8)	0.168
Pain						

Baseline	14	58.3 ± 31.8	11	42.4 ± 33.6	-15.9 (-43.1 – 11.2)	0.239
2 cycles	9	51.8 ± 37.6	8	29.1 ± 23.1		
4 cycles	7	50.0 ± 30.4	8	24.9 ± 21.8		
6 cycles	6	30.5 ± 35.6	5	30.0 ± 27.3	-0.5 (-44.6 – 43.5)	0.978
P Baseline - 6 cycles		0.141		0.314		
Δ Baseline - 6 cycles	6	-13.8 ± 19.4	5	-16.6 ± 37.2	-2.7 (-42.1 – 36.6)	0.877
Dyspnea						
Baseline	13	38.4 ± 44.8	11	33.3 ± 25.8	-5.1 (-35.7 – 25.5)	0.730
2 cycles	7	61.9 ± 35.6	8	33.3 ± 25.1		
4 cycles	7	14.2 ± 17.8	8	33.3 ± 39.8		
6 cycles	6	22.2 ± 40.3	5	33.3 ± 40.8	11.1 (-44.4 – 66.6)	0.662
P Baseline - 6 cycles		0.999		0.999		
Δ Baseline - 6 cycles	6	0 ± 21.0	5	0 ± 33.3	0 (-37.2 – 37.2)	0.999
Insomnia						
Baseline	14	64.2 ± 30.5	11	57.5 ± 39.6	-6.7 (-35.7 – 22.3)	0.637
2 cycles	8	45.8 ± 43.4	8	37.5 ± 45.2		
4 cycles	7	23.8 ± 31.7	8	37.4 ± 33.0		
6 cycles	6	38.8 ± 38.9	5	53.3 ± 29.8	14.4 (-33.7 – 62.6)	0.515
P Baseline - 6 cycles		0.296		0.838		
Δ Baseline - 6 cycles	6	-16.6 ± 34.9	5	-6.6 ± 68.3	9.9 (-61.8 – 81.8)	0.760
Appetite loss						
Baseline	13	46.1 ± 34.7	11	48.4 ± 34.5	2.3 (-27.1 – 31.7)	0.871
2 cycles	9	40.7 ± 27.7	8	41.6 ± 29.5		
4 cycles	7	52.3 ± 37.7	8	33.3 ± 17.8		
6 cycles	6	38.8 ± 32.7	5	59.9 ± 36.5	21.1 (-26.1 – 68.3)	0.338

P Baseline - 6 cycles		0.175		0.294		
Δ Baseline - 6 cycles	6	11.1 ± 17.2	5	26.6 ± 49.4	15.5 (-32.8 – 64.0)	0.486
Constipation						
Baseline	14	38.0 ± 31.6	11	21.2 ± 26.9	-16.8 (-41.6 – 7.8)	0.172
2 cycles	9	55.5 ± 40.8	8	54.1 ± 24.8		
4 cycles	7	23.8 ± 25.1	8	33.3 ± 35.6		
6 cycles	6	16.6 ± 40.8	5	0 ± 0	-16.6 (-58.3 – 25.0)	0.389
P Baseline - 6 cycles		0.296		0.208		
Δ Baseline - 6 cycles	6	-16.6 ± 34.9	5	-20.0 ± 29.8	-3.3 (-48.2 – 41.5)	0.870
Diarrhea						
Baseline	13	15.3 ± 32.2	11	12.1 ± 30.8	-3.2 (-30.1 – 23.5)	0.803
2 cycles	9	40.7 ± 40.0	8	8.3 ± 15.4		
4 cycles	7	23.8 ± 25.1	8	8.3 ± 15.4		
6 cycles	6	16.6 ± 18.2	5	26.6 ± 36.5	10.0 (-34.6 – 54.6)	0.599
P Baseline - 6 cycles		0.999		0.999		
Δ Baseline - 6 cycles	6	-0.0 ± 36.5	5	0.0 ± 62.3	0 (-68.0 – 68.0)	0.999
Financial difficulties						
Baseline	14	76.1 ± 30.4	11	51.5 ± 34.5	-24.6 (-51.5 – 2.2)	0.071
2 cycles	9	66.6 ± 28.8	7	52.3 ± 26.2		
4 cycles	7	57.1 ± 25.1	8	54.1 ± 43.4		
6 cycles	6	72.2 ± 32.7	5	80.0 ± 50.5	7.7 (-49.2 – 64.7)	0.765
P Baseline - 6 cycles		0.576		0.468		
Δ Baseline - 6 cycles	6	-11.1 ± 45.5	5	19.9 ± 55.7	31.1 (-37.8 – 100)	0.334
Dyspnea 2						
Baseline	13	37.6 ± 24.6	11	36.3 ± 29.0	-1.2 (-23.9 – 21.4)	0.911

2 cycles	9	37.0 ± 26.0	8	24.9 ± 27.0		
4 cycles	7	20.6 ± 25.1	8	26.3 ± 28.4		
6 cycles	6	24.0 ± 38.7	5	20.0 ± 29.8	-4.0 (-52.1 – 43.9)	0.852
P Baseline - 6 cycles		0.363		0.108		
Δ Baseline - 6 cycles	6	-5.5 ± 13.6	5	-31.1 ± 33.7	-25.5 (-66.6 – 15.5)	0.171
**Coughing**						
Baseline	13	56.4 ± 34.3	11	54.5 ± 22.4	-1.8 (-26.2 – 22.4)	0.875
2 cycles	9	40.7 ± 46.4	8	37.4 ± 21.3		
4 cycles	7	28.5 ± 29.9	8	37.5 ± 37.5		
6 cycles	6	50.0 ± 34.9	5	19.9 ± 18.2	-30.0 (-69.3 – 9.3)	0.119
P Baseline - 6 cycles		0.121		**0.034**		
Δ Baseline - 6 cycles	6	0 ± 29.8	5	-33.3 ± 23.5	-33.3 (-70.6 – 3.9)	0.074
Haemoptysis						
Baseline	13	2.5 ± 9.2	11	3.0 ± 10.0	0.4 (-7.7 – 8.6)	0.907
2 cycles	9	0 ± 0	8	0 ± 0		
4 cycles	7	0 ± 0	8	8.3 ± 15.4		
6 cycles	6	0 ± 0	5	0 ± 0	n/a	n/a
P Baseline - 6 cycles		n/a		0.374		
Δ Baseline - 6 cycles	6	0 ± 0	5	-6.6 ± 14.9	-6.6 (-25.1 – 11.8)	0.374
Sore mouth						
Baseline	13	15.3 ± 32.2	11	21.2 ± 34.2	5.8 (-22.3 – 34.0)	0.672
2 cycles	9	14.8 ± 33.7	8	4.1 ± 11.7		
4 cycles	7	4.7 ± 12.5	8	8.3 ± 15.4		
6 cycles	6	11.1 ± 17.2	5	26.6 ± 27.8	15.5 (-15.3 – 46.4)	0.285
P Baseline - 6 cycles		0.363		0.749		

Δ Baseline - 6 cycles	6	-11.1 ± 27.2	5	6.6 ± 43.4	17.7 (-30.6 – 66.2)	0.428
Dysphagia						
Baseline	13	35.8 ± 44.0	11	21.2 ± 22.4	-14.6 (-43.9 – 14.5)	0.306
2 cycles	8	29.1 ± 33.0	8	41.6 ± 38.8		
4 cycles	7	9.5 ± 16.2	8	25.0 ± 29.5		
6 cycles	6	11.1 ± 27.2	5	46.6 ± 50.5	35.5 (-26.3 – 97.4)	0.209
P Baseline - 6 cycles		0.363		0.337		
Δ Baseline - 6 cycles	6	-16.6 ± 40.8	5	26.6 ± 54.7	43.3 (-21.7 – 108.4)	0.166
Peripheral neuropathy						
Baseline	13	25.6 ± 36.3	11	21.2 ± 30.8	-4.4 (-32.8 – 24.0)	0.750
2 cycles	9	51.8 ± 47.4	7	42.8 ± 46.0		
4 cycles	7	42.8 ± 31.7	8	37.5 ± 37.5		
6 cycles	6	55.5 ± 40.3	5	53.3 ± 50.5	-2.2 (-64.1 – 59.6)	0.937
P Baseline - 6 cycles		**0.042**		0.070		
Δ Baseline - 6 cycles	6	27.7 ± 25.0	5	40.0 ± 36.5	12.2 (-33.4 – 57.9)	0.546
Alopecia						
Baseline	13	28.2 ± 40.4	11	12.1 ± 16.8	-16.0 (-43.2 – 11.0)	0.232
2 cycles	9	37.0 ± 42.3	8	58.3 ± 42.7		
4 cycles	7	38.0 ± 35.6	8	62.5 ± 27.8		
6 cycles	6	77.7 ± 40.3	5	73.3 ± 27.8	-4.4 (-52.8 – 44.0)	0.840
P Baseline - 6 cycles		0.178		**0.009**		
Δ Baseline - 6 cycles	6	50.0 ± 78.1	5	60.0 ± 27.8	10.0 (-72.4 – 92.4)	0.780
Pain in chest						

Baseline	13	25.6 ± 30.8	11	27.2 ± 25.0	1.6 (-22.4 – 25.7)	0.890
2 cycles	8	45.8 ± 39.5	7	42.8 ± 46.0		
4 cycles	7	19.0 ± 26.2	8	24.9 ± 23.5		
6 cycles	6	22.2 ± 40.3	5	40.0 ± 27.8	17.7 (-30.6 – 66.2)	0.428
P Baseline - 6 cycles		0.363		0.621		
Δ Baseline - 6 cycles	6	0 ± 0	5	6.6 ± 27.8	6.6 (-27.9 – 41.3)	0.621
Pain in arm or shoulder						
Baseline	13	33.3 ± 30.4	11	18.1 ± 22.9	-15.1 (-38.3 – 8.0)	0.189
2 cycles	9	55.5 ± 40.8	8	20.8 ± 35.3		
4 cycles	7	23.8 ± 25.1	8	16.6 ± 17.8		
6 cycles	6	27.7 ± 38.9	5	33.3 ± 23.5	5.5 (-39.6 – 50.7)	0.787
P Baseline - 6 cycles		0.999		0.208		
Δ Baseline - 6 cycles	6	-11.1 ± 27.2	5	20.0 ± 29.8	31.1 (-8.6 – 70.8)	0.110
Pain in other parts						
Baseline	13	35.8 ± 21.3	11	33.3 ± 39.4	-2.5 (-28.8 – 23.7)	0.841
2 cycles	8	29.1 ± 33.0	8	16.6 ± 17.8		
4 cycles	6	27.7 ± 13.6	8	20.8 ± 17.2		
6 cycles	6	16.6 ± 18.2	5	33.3 ± 0.0	16.6 (-2.4 – 35.8)	0.076
P Baseline - 6 cycles		0.203		0.070		
Δ Baseline - 6 cycles	6	-16.6 ± 27.8	5	19.9 ± 18.2	36.6 (3.6 – 69.6)	**0.033**

Supplementary Table 3. Health-related quality of life among patients without depression at baseline

Health related quality of life	n	C (mean ± DE)	n	EPC (mean ± DE)	**Mean difference (95% CI)**	p (t student)
Global health status						
Baseline	35	57.3 ± 23.2	34	70.3 ± 22.9	12.9 (1.8 – 24.0)	0.023
2 cycles	22	64.3 ± 17.2	59	67.8 ± 27.8		
4 cycles	22	57.9 ± 23.3	26	66.9 ± 25.3		
6 cycles	21	59.9 ± 21.6	25	64.0 ± 22.6	4.0 (-9.1 – 17.3)	0.538
P Baseline - 6 cycles		0.204		0.318		
Δ Baseline - 6 cycles	21	-5.9 ± 20.7	25	-4.9 ± 24.5	0.9 (-12.7 – 14.6)	0.889
Physical functioning						
Baseline	35	71.4 ± 24.8	34	75.8 ± 20.8	4.4 (-6.5 – 15.4)	0.423
2 cycles	23	73.9 ± 22.9	30	77.1 ± 18.6		
4 cycles	22	73.0 ± 23.8	26	74.1 ± 22.4		
6 cycles	21	78.0 ± 19.3	25	78.9 ± 15.8	0.8 (-9.6 – 11.2)	0.872
P Baseline - 6 cycles		0.479		0.530		
Δ Baseline - 6 cycles	21	3.4 ± 22.1	25	3.4 ± 27.2	-0.02 (-14.9 – 14.9)	0.997
Role functioning						
Baseline	35	62.3 ± 32.4	33	76.7 ± 26.9	14.3 (-0.1 – 28.8)	0.052
2 cycles	23	72.4 ± 25.4	30	71.6 ± 30.3		
4 cycles	22	69.6 ± 33.9	26	73.7 ± 29.1		
6 cycles	21	76.1 ± 22.0	25	78.0 ± 22.4	1.8 (-11.4 – 15.0)	0.785
P Baseline - 6 cycles		0.370		0.648		
Δ Baseline - 6 cycles	20	1.6 ± 25.8	22	6.8 ± 36.2	5.1 (-14.6 – 24.9)	0.602
Emotional functioning						
Baseline	35	76.4 ± 18.9	34	75.7 ± 18.7	-0.6 (-9.7 – 8.3)	0.879
2 cycles	22	74.6 ± 19.8	29	77.0 ± 23.7		
4 cycles	22	77.2 ± 21.6	26	74.6 ± 25.6		
6 cycles	21	80.9 ± 19.0	25	80.3 ± 17.6	-0.6 (-11.5 – 10.2)	0.910

P Baseline - 6 cycles		0.694		0.110		
Δ Baseline - 6 cycles	21	2.3 ± 27.2	25	7.0 ± 21.0	4.6 (-9.7 – 18.9)	0.520
Cognitive functioning						
Baseline	35	89.5 ± 12.8	34	87.7 ± 16.5	-1.7 (-8.8 – 5.3)	0.619
2 cycles	22	84.0 ± 18.1	29	83.9 ± 23.7		
4 cycles	22	86.3 ± 13.2	25	80.6 ± 23.9		
6 cycles	21	80.1 ± 20.8	25	85.3 ± 20.0	5.1 (-6.9 – 17.3)	0.396
P Baseline - 6 cycles		0.018		0.705		
Δ Baseline - 6 cycles	21	-11.9 ± 21.1	25	-1.9 ± 26.0	9.9 (-4.3 – 24.1)	0.170
Social functioning						
Baseline	35	80.4 ± 24.0	34	76.9 ± 28.1	-3.5 (-16.0 – 9.0)	0.579
2 cycles	22	66.6 ± 24.1	29	77.5 ± 22.3		
4 cycles	22	75.0 ± 25.5	26	76.9 ± 17.6		
6 cycles	21	72.2 ± 25.9	25	68.0 ± 30.7	-4.2 (-21.3 – 12.9)	0.622
P Baseline - 6 cycles		0.310		0.156		
Δ Baseline - 6 cycles	21	-7.1 ± 31.4	25	-11.3 ± 38.7	-4.1 (-25.4 – 17.0)	0.693
Fatigue						
Baseline	35	39.9 ± 24.8	34	33.0 ± 21.4	-6.9 (-18.1 – 4.1)	0.216
2 cycles	23	36.7 ± 21.8	30	37.4 ± 26.8		
4 cycles	22	37.3 ± 22.6	26	37.1 ± 25.5		
6 cycles	21	32.8 ± 15.5	25	32.4 ± 17.5	-0.3 (-10.2 – 9.5)	0.942
P Baseline - 6 cycles		0.599		0.784		
Δ Baseline - 6 cycles	21	-3.1 ± 27.2	25	-1.3 ± 24.0	1.8 (-13.4 – 17.0)	0.809
Nausea and vomiting						
Baseline	35	12.3 ± 17.7	34	10.7 ± 15.2	-1.5 (-9.5 – 6.3)	0.691
	23	25.3 ± 24.5	30	19.4 ± 27.3		
4 cycles	21	19.0 ± 22.5	26	19.2 ± 24.8		
6 cycles	21	18.2 ± 25.2	25	13.3 ± 15.2	-4.9 (-17.0 – 7.2)	0.419

P Baseline - 6 cycles		0.637		0.866		
Δ Baseline - 6 cycles	21	3.9 ± 37.9	25	0.6 ± 19.5	-3.3 (-20.8 – 14.2)	0.706
Pain						
Baseline	35	31.4 ± 26.4	34	33.8 ± 27.6	2.3 (-10.6 – 15.3)	0.714
2 cycles	23	28.2 ± 24.8	29	29.8 ± 27.5		
4 cycles	22	33.3 ± 27.2	26	33.9 ± 33.8		
6 cycles	21	26.1 ± 25.0	25	31.3 ± 26.0	5.1 (-10.1 – 20.4)	0.501
P Baseline - 6 cycles		0.452		0.574		
Δ Baseline - 6 cycles	21	4.7 ± 28.4	25	-3.3 ± 29.2	-8.0 (-25.3 – 9.1)	0.349
Dyspnea						
Baseline	35	21.9 ± 27.9	34	22.5 ± 24.2	0.6 (-11.9 -13.2)	0.919
2 cycles	21	23.8 ± 21.4	30	22.2 ± 31.9		
4 cycles	22	24.2 ± 21.0	26	20.5 ± 28.4		
6 cycles	21	19.0 ± 22.5	25	15.9 ± 21.7	-3.0. (-16.2 – 10.1)	0.644
P Baseline - 6 cycles		0.329		0.203		
Δ Baseline - 6 cycles	21	6.3 ± 29.0	25	-6.6 ± 25.4	-13.0 (-29.2 – 3.1)	0.113
Insomnia						
Baseline	35	41.9 ± 35.5	34	33.3 ± 30.7	-8.5 (-24.5 – 7.4)	0.288
2 cycles	22	34.8 ± 29.9	30	19.9 ± 25.6		
4 cycles	22	33.3 ± 35.6	26	19.2 ± 26.9		
6 cycles	21	28.5 ± 30.3	25	21.3 ± 27.0	-7.2 (-24.2 – 9.8)	0.397
P Baseline - 6 cycles		0.634		0.107		
Δ Baseline - 6 cycles	21	-4.7 ± 45.0	25	-12.0 ± 35.8	-7.2 (-31.2 – 16.8)	0.547
Appetite loss						
Baseline	35	26.6 ± 28.9	34	28.4 ± 33.9	1.7 (-13.3 – 16.9)	0.817
2 cycles	23	30.4 ± 31.6	30	28.8 ± 35.8		
4 cycles	22	34.8 ± 34.8	26	26.9 ± 31.2		
6 cycles	21	22.2 ± 24.3	25	29.3 ± 24.1	7.1 (-7.3 – 21.5)	0.327

P Baseline - 6 cycles		0.847		0.746		
Δ Baseline - 6 cycles	21	-1.5 ± 37.2	25	-2.6 ± 40.7	-1.0 (-24.4 – 22.2)	0.926
Constipation						
Baseline	35	21.9 ± 30.1	34	24.5 ± 33.1	2.6 (-12.6 – 17.8)	0.734
2 cycles	23	20.2 ± 26.0	30	9.9 ± 19.8		
4 cycles	22	21.2 ± 30.0	26	20.5 ± 28.4		
6 cycles	21	14.2 ± 19.9	25	19.9 ± 25.4	5.7 (-8.0 – 19.4)	0.408
P Baseline - 6 cycles		0.428		0.380		
Δ Baseline - 6 cycles	21	-6.3 ± 35.9	25	-6.6 ± 37.2	-0.3 (-22.1 – 21.5)	0.977
Diarrhea						
Baseline	35	3.8 ± 10.7	34	10.7 ± 19.6	6.9 (-0.7 – 14.6)	0.074
2 cycles	22	27.2 ± 33.5	29	18.3 ± 26.1		
4 cycles	22	13.6 ± 19.6	26	8.9 ± 22.2		
6 cycles	21	14.2 ± 16.9	25	9.3 ± 15.2	-4.9 (-14.5 – 4.6)	0.302
P Baseline - 6 cycles		0.135		0.603		
Δ Baseline - 6 cycles	21	7.9 ± 23.3	25	-2.6 ± 25.3	-10.6 (-25.1 – 3.9)	0.150
Financial difficulties						
Baseline	35	53.3. ± 33.5	34	56.8 ± 36.2	3.5 (-13.2 – 20.3)	0.676
2 cycles	22	63.6 ± 30.7	29	51.7 ± 35.1		
4 cycles	21	52.3 ± 29.0	26	57.6 ± 32.0		
6 cycles	21	65.0 ± 28.8	25	62.6 ± 41.1	-2.4 (-23.9 – 19.1)	0.822
P Baseline - 6 cycles		0.448		0.548		
Δ Baseline - 6 cycles	21	7.9 ± 47.0	25	5.3 ± 43.7	-2.6 (-29.6 – 24.4)	0.847
Dyspnea 2						
Baseline	35	25.7 ± 23.9	34	21.2 ± 22.2	-4.4 (-15.5 – 6.6)	0.425
2 cycles	23	27.5 ± 21.9	30	23.7 ± 26.0		
4 cycles	22	24.2 ± 23.1	26	21.7 ± 24.8		
6 cycles	21	19.5 ± 16.4	25	14.2 ± 19.1	-5.3 (-16.0 – 5.3)	0.319

P Baseline - 6 cycles		0.827		0.391		
Δ Baseline - 6 cycles	21	1.0 ± 21.9	25	-4.4 ± 25.4	-5.5 (-19.7 – 8.7)	0.441
Coughing						
Baseline	35	52.3 ± 27.1	34	36.2 ± 26.4	-16.1 (-28.9 – (-3.2))	0.015
2 cycles	23	40.5 ± 30.0	30	35.5 ± 27.5		
4 cycles	22	31.8 ± 24.0	26	26.9 ± 24.9		
6 cycles	21	33.3 ± 25.8	25	18.6 ± 19.4	-14.6 (-28.1 – (-1.2))	0.033
P Baseline - 6 cycles		0.130		0.015		
Δ Baseline - 6 cycles	21	-11.1 ± 32.2	25	-13.3 ± 25.4	-2.22 (-19.3 – 14.9)	0.795
Haemoptysis						
Baseline	35	13.3 ± 20.1	34	4.9 ± 11.9	-8.4 (-16.3 – (-0.4))	0.038
2 cycles	23	4.3 ± 11.4	30	1.1 ± 6.0		
4 cycles	22	3.0 ± 9.8	26	1.2 ± 6.5		
6 cycles	21	6.3 ± 22.6	25	3.9 ± 11.0	-2.3 (-12.6 – 7.9)	0.649
P Baseline - 6 cycles		0.296		0.999		
Δ Baseline - 6 cycles	21	-6.3 ± 27.1	25	0 ± 13.6	6.3 (-6.9 – 19.6)	0.338
Sore mouth						
Baseline	35	1.9 ± 7.8	34	5.8 ± 15.2	3.9 (-1.8 – 9.7)	0.177
2 cycles	23	4.3 ± 11.4	30	6.6 ± 20.3		
4 cycles	22	7.5 ± 14.2	26	12.8 ± 28.4		
6 cycles	21	3.1 ± 10.0	25	5.3 ± 12.4	2.1 (-4.6 -8.9)	0.527
P Baseline - 6 cycles		0.999		0.770		
Δ Baseline - 6 cycles	21	0 ± 10.5	25	-1.3 ± 22.5	-1.3 (-11.5 – 8.9)	0.794
Dysphagia						
Baseline	35	9.5 ± 20.7	34	8.8 ± 17.0	-0.7 (-9.8 – 8.4)	0.879
2 cycles	22	13.6 ± 24.4	30	20.0 ± 32.2		
4 cycles	22	7.5 ± 17.6	26	11.5 ± 26.5		
6 cycles	21	4.7 ± 15.9	25	13.3 ± 21.5	8.5 (-2.8 – 20.0)	0.138

P Baseline - 6 cycles		0.329		0.161		
Δ Baseline - 6 cycles	21	-1.5 ± 7.2	25	5.3 ± 18.4	6.9 (-1.2 – 15.1)	0.095
Peripheral neuropathy						
Baseline	35	16.1 ± 24.7	34	26.4 ± 63.9	10.2 (-12.9 – 33.4)	0.379
2 cycles	23	36.2 ± 34.6	30	24.4 ± 30.2		
4 cycles	22	34.8 ± 29.9	26	26.9 ± 23.1		
6 cycles	21	41.2 ± 29.6	25	27.9 ± 28.3	-13.2 (-30.5 – 3.9)	0.129
P Baseline - 6 cycles		0.001		0.162		
Δ Baseline - 6 cycles	21	23.8 ± 28.1	25	10.6 ± 36.9	-13.1 (-32.5 – 6.2)	0.178
Alopecia						
Baseline	35	18.0 ± 32.6	34	22.5 ± 31.4	4.4 (-10.9 – 19.8)	0.566
2 cycles	23	56.5 ± 40.7	30	66.6 ± 40.1		
4 cycles	22	60.6 ± 33.5	26	46.1 ± 45.2		
6 cycles	21	63.4 ± 44.6	24	40.2 ± 45.0	-23.2 (-50.2 – 3.7)	0.090
P Baseline - 6 cycles		0.003		0.075		
Δ Baseline - 6 cycles	21	44.4 ± 60.8	24	19.4 ± 50.9	-25.0 (-58.6 – 8.6)	0.141
Pain in chest						
Baseline	35	25.7 ± 30.3	34	20.5 ± 32.8	-5.1 (-20.3 – 10.0)	0.503
2 cycles	22	21.2 ± 26.3	30	19.9 ± 29.8		
4 cycles	22	19.6 ± 22.2	25	14.6 ± 25.6		
6 cycles	21	17.4 ± 27.1	25	21.3 ± 27.0	3.8 (-12.2 – 20.0)	0.631
P Baseline - 6 cycles		0.693		0.307		
Δ Baseline - 6 cycles	21	-3.1 ± 36.3	25	6.6 ± 31.9	9.8 (-10.4 – 30.1)	0.334
Pain in arm or shoulder						
Baseline	34	19.6 ± 23.3	34	24.5 ± 27.5	4.9 (-7.4 – 17.2)	0.432
2 cycles	23	20.2 ± 27.9	30	8.8 ± 21.3		
4 cycles	22	16.6 ± 24.6	26	11.5 ± 20.9		
6 cycles	21	26.9 ± 27.1	25	22.6 ± 28.4	-4.3 (-20.9 – 12.2)	0.603

P Baseline - 6 cycles		0.110		0.840		
Δ Baseline - 6 cycles	21	11.1 ± 30.4	25	1.3 ± 32.6	-9.7 (-28.6 – 9.0)	0.302
Pain in other parts						
Baseline	35	29.5 ± 25.2	33	32.3 ± 24.2	2.7 (-9.2 – 14.8)	0.643
2 cycles	21	23.8 ± 21.4	28	26.1 ± 18.9		
4 cycles	21	28.5 ± 24.2	24	22.2 ± 16.0		
6 cycles	20	24.9 ± 21.2	23	24.6 ± 20.6	-0.3 (-13.2 – 12.5)	0.955
P Baseline - 6 cycles		0.825		0.110		
Δ Baseline - 6 cycles	20	-1.6 ± 33.2	23	-8.6 ± 25.0	-7.0 (-25.0 – 10.9)	0.435

Supplementary Table 4. Health-related quality of life among patients with depression at baseline

Health related quality of life	n	**C** (mean ± DE)	n	**EPC** (mean ± DE)	**Mean difference (95% CI)**	p (t student)
Global health status						
Baseline	23	40.2 ± 27.0	24	42.0 ± 20.0	1.7 (-12.1 – 15.7)	0.796
2 cycles	14	60.1 ± 21.4	12	48.6 ± 23.5		
4 cycles	11	52.2 ± 26.8	13	57.6 18.4		
6 cycles	9	54.6 ± 27.0	11	50.7 ± 27.7	-3.8 (-29.7 – 22.0)	0.757
P Baseline - 6 cycles		0.071		0.945		
Δ Baseline - 6 cycles	9	24.0 ± 34.7	11	0.7 ± 35.2	-23.3 (-56.3 – 9.7)	0.156
Physical functioning						
Baseline	23	47.2 ± 28.9	24	52.2 ± 23.2	4.9 (-10.4 – 20.3)	0.519
2 cycles	14	62.8 ± 27.6	14	57.6 ± 22.2		
4 cycles	11	64.8 ± 25.4	13	54.3 ± 19.5		
6 cycles	9	62.2 ± 30.5	11	40.6 ± 31.3	-21.6 (-50.8 – 7.6)	0.138
P Baseline - 6 cycles		0.907		0.171		
Δ Baseline - 6 cycles	9	0.7 ± 18.3	11	-15.7 ± 35.4	-16.4 (-43.9 – 11.0)	0.224

Role functioning						
Baseline	23	39.1 ± 39.1	24	42.3 ± 29.4	3.2 (-17.0 – 23.5)	0.750
2 cycles	14	60.7 ± 40.0	14	47.6 ± 30.5		
4 cycles	11	56.0 ± 34.3	13	51.2 ± 39.9		
6 cycles	9	55.5 ± 35.3	11	48.4 ± 35.3	-7.0 (-40.4 – 26.2)	0.662
P Baseline - 6 cycles		0.738		0.896		
Δ Baseline - 6 cycles	8	-16.6 ± 12.5	10	-6.6 ± 55.1	10.0 (-32.3 – 52.3)	0.624
Emotional functioning						
Baseline	22	44.3 ± 28.2	24	59.3 ± 22.2	15.0 (0.0 – 30.1)	0.050
2 cycles	14	71.4 ± 29.1	12	72.2 ± 24.4		
4 cycles	11	67.4 ± 19.5	13	76.2 ± 16.2		
6 cycles	9	61.1 ± 33.3	11	59.8 ± 24.9	-1.2 (-28.6 – 26.1)	0.924
P Baseline - 6 cycles		0.198		0.305		
Δ Baseline - 6 cycles	9	14.8 ± 31.6	11	-8.3 ± 25.5	-23.1 (-49.9 – 3.7)	0.087
Cognitive functioning						
Baseline	23	55.7 ± 35.7	24	72.9 ± 23.9	17.1 (-0.9 – 35.1)	0.062
2 cycles	14	69.0 ± 28.3	12	84.7 ± 18.0		
4 cycles	11	69.6 ± 23.3	13	74.3 ± 24.1		
6 cycles	9	70.3 ± 34.1	11	69.6 ± 37.8	-0.6 (-34.9 – 33.5)	0.967
P Baseline - 6 cycles		0.198		0.684		
Δ Baseline - 6 cycles	9	14.8 ± 31.6	11	-6.0 ± 47.8	-20.8 (-60.0 – 18.2)	0.277
Social functioning						
Baseline	23	52.8 ± 37.8	24	53.4 ± 34.0	0.5 (-20.5 – 21.6)	0.957
2 cycles	14	55.9 ± 33.0	12	62.4 ± 37.0		
4 cycles	11	53.0 ± 23.3	13	46.1 ± 34.1		
6 cycles	9	61.1 ± 33.3	11	59.0 ± 39.6	-2.0 (-36.9 – 32.9)	0.905
P Baseline - 6 cycles		0.773		0.769		
Δ Baseline - 6 cycles	9	5.5 ± 55.9	11	-4.5 ± 50.0	-10.1 (-59.8 – 39.6)	0.675

Fatigue						
Baseline	23	61.8 ± 26.7	24	60.1 ± 22.2	-1.6 (-16.0 – 12.7)	0.819
2 cycles	14	46.8 ± 31.4	14	49.2 ± 26.7		
4 cycles	11	42.4 ± 25.2	13	47.0 ± 18.2		
6 cycles	9	40.7 ± 28.3	11	59.5 ± 33.0	18.8 (-10.4 – 48.1)	0.193
P Baseline - 6 cycles		0.170		0.922		
Δ Baseline - 6 cycles	9	-16.0 ± 31.9	11	1.0 ± 33.1	17.0 (-13.7 – 47.8)	0.260
Nausea and vomiting						
Baseline	22	39.3 ± 35.8	23	19.5 ± 19.8	-19.8 (-37.5 – (-2.1))	0.029
2 cycles	14	35.7 ± 31.2	14	23.8 ± 24.2		
4 cycles	11	18.1 ± 26.3	13	28.2 ± 26.6		
6 cycles	9	35.1 ± 39.4	11	18.1 ± 21.6	-17.0 (-46.1 – 12.1)	0.068
P Baseline - 6 cycles		0.479		0.440		
Δ Baseline - 6 cycles	9	-9.2 ± 37.3	11	-6.0 ± 25.0	3.1 (-26.1 – 32.5)	0.822
Pain						
Baseline	23	52.1 ± 35.2	24	43.0 ± 29.8	-9.1 (-28.2 – 10.0)	0.343
2 cycles	14	51.1 ± 32.3	13	33.3 ± 22.5		
4 cycles	11	34.8 ± 21.6	13	33.3 ± 30.4		
6 cycles	9	48.1 ± 29.3	11	28.7 ± 27.9	-19.3 (-46.3 – 7.6)	0.150
P Baseline - 6 cycles		0.211		0.120		
Δ Baseline - 6 cycles	9	12.9 ± 28.6	11	-16.6 ± 32.4	-29.6 (-58.7 – 0.5)	**0.046**
Dyspnea						
Baseline	21	49.2 ± 40.3	24	37.4 ± 29.9	-11.7 (-32.8 – 9.4)	0.271
2 cycles	14	26.1 ± 39.6	14	35.7 ± 33.2		
4 cycles	11	9.0 ± 15.5	13	30.7 ± 31.8		
6 cycles	9	14.8 ± 33.7	11	36.3 ± 37.8	21.5 (-12.5 – 55.6)	0.201
P Baseline - 6 cycles		0.080		0.999		
Δ Baseline - 6 cycles	8	-24.9 ± 34.5	11	0 ± 29.8	24.9 (-6.2 – 56.2)	0.109

Insomnia						
Baseline	23	52.1 ± 38.6	24	52.7 ± 30.9	0.6 (-19.9 – 21.1)	0.953
2 cycles	14	35.7 ± 38.0	14	42.8 ± 27.5		
4 cycles	11	30.3 ± 31.4	13	30.7 ± 21.3		
6 cycles	9	37.0 ± 30.9	11	33..3 ± 21.0	-3.7 (-28.1 – 20.7)	0.754
P Baseline - 6 cycles		0.799		0.046		
Δ Baseline - 6 cycles	9	-3.7 ± 42.3	11	-21.2 ± 30.8	-17.5 (-51.8 – 16.8)	0.298
Appetite loss						
Baseline	22	57.5 ± 34.3	24	50 ± 29.4	-7.5 (-26.5 – 11.4)	0.426
2 cycles	14	28.5 ± 34.2	14	57.1 ± 33.1		
4 cycles	11	45.4 ± 34.2	13	43.5 ± 31.5		
6 cycles	9	51.8 ± 37.6	11	45.4 ± 34.2	-6.3 (-40.2 – 27.4)	0.696
P Baseline - 6 cycles		0.708		0.846		
Δ Baseline - 6 cycles	9	7.4 ± 57.1	11	3.0 ± 50.4	-4.3 (-54.9 – 46.1)	0.858
Constipation						
Baseline	23	27.5 ± 31.2	24	27.7 ± 27.2	0.2 (-16.9 – 17.4)	0.977
2 cycles	14	42.8 ± 37.9	14	38.0 ± 34.2		
4 cycles	11	21.2 ± 26.9	13	35.8 ± 28.7		
6 cycles	9	29.6 ± 35.1	11	21.2 ± 26.9	-8.4 (-37.5 – 20.7)	0.552
P Baseline - 6 cycles		0.999		0.999		
Δ Baseline - 6 cycles	9	0 ± 40.8	11	0 ± 33.3	0 (-34.7 – 34.7)	0.999
Diarrhea						
Baseline	22	21.2 ± 33.4	24	12.4 ± 23.6	-8.7 (-25.8 – 8.3)	0.310
2 cycles	14	19.0 ± 31.2	12	8.3 ± 15.0		
4 cycles	11	12.1 ± 16.8	13	10.2 ± 16.0		
6 cycles	9	11.1 ± 23.5	11	18.1 ± 22.9	7.0 (-14.8 – 28.9)	0.507
P Baseline - 6 cycles		0.141		0.821		
Δ Baseline - 6 cycles	9	-22.2 ± 40.8	11	3.0 ± 43.3	25.2 (-14.6 – 65.1)	0.200

Financial difficulties						
Baseline	23	62.3 ± 35.2	24	59.7 ± 36.7	-2.5 (-23.7 – 18.5)	0.806
2 cycles	14	69.0 ± 24.3	11	66.6 ± 33.3		
4 cycles	11	72.7 ± 25.0	13	61.5 ± 35.6		
6 cycles	9	66.6 ± 33.3	11	75.7 ± 33.6	9.0 (-22.5 – 40.7)	0.554
P Baseline - 6 cycles		0.512		0.588		
Δ Baseline - 6 cycles	9	7.4 ± 32.3	11	6.0 ± 35.9	-1.3 (-33.8 – 31.5)	0.932
Dyspnea 2						
Baseline	22	35.3 ± 23.9	23	39.1 ± 28.2	3.7 (-11.9 – 19.5)	0.631
2 cycles	14	24.6 ± 25.8	14	36.5 ± 28.3		
4 cycles	11	16.1 ± 20.7	12	33.3 ± 25.9		
6 cycles	9	23.4 ± 32.6	11	32.3 ± 32.7	8.8 (-22.0 – 39.7)	0.554
P Baseline - 6 cycles		0.384		0.650		
Δ Baseline - 6 cycles	9	-6.1 ± 20.1	11	-4.0 ± 28.6	2.1 (-21.6 – 25.9)	0.853
**Coughing**						
Baseline	22	59.0 ± 32.4	23	47.8 ± 33.0	-11.2 (-30.9 – 8.4)	0.255
2 cycles	14	35.7 ± 33.2	14	35.7 ± 30.5		
4 cycles	11	33.3 ± 21.0	12	30.5 ± 22.2		
6 cycles	9	33.3 ± 28.8	11	24.2 ± 26.2	-9.0 (-34.9 – 16.8)	0.470
P Baseline - 6 cycles		0.051		0.132		
Δ Baseline - 6 cycles	9	-18.5 ± 24.2	11	-21.2 ± 42.8	-2.6 (-36.5 – 31.1)	0.869
Haemoptysis						
Baseline	22	19.6 ± 31.9	23	5.7 ± 16.3	-13.8 (-29.0 – 1.2)	0.072
2 cycles	14	2.3 ± 8.9	14	2.3 ± 8.9		
4 cycles	11	3.0 ± 10.0	12	2.7 ± 9.6		
6 cycles	9	7.4 ± 22.2	11	0 ± 0	-7.4 (-24.4 – 9.6)	0.347
P Baseline - 6 cycles		0.559		0.192		
Δ Baseline - 6 cycles	9	-7.4 ± 36.4	11	-9.0 ± 21.5	-1.6 (-29.1 – 25.8)	0.899

Sore mouth						
Baseline	22	12.1 ± 26.3	23	15.9 ± 31.5	3.8 (-13.6 – 21.3)	0.662
2 cycles	14	14.2 ± 31.2	14	11.9 ± 16.5		
4 cycles	11	0.0 ± 0	12	13.8 ± 22.2		
6 cycles	9	11.1 ± 16.6	11	12.1 ± 22.4	1.0 (-17.9 – 19.9)	0.912
P Baseline - 6 cycles		0.512		0.506		
Δ Baseline - 6 cycles	9	-7.4 ± 32.3	11	-6.0 ± 29.1	1.3 (-27.5 – 30.2)	0.923
Dysphagia						
Baseline	22	34.8 ± 37.7	23	17.3 ± 24.3	-17.4 (-36.4 – 1.5)	0.071
2 cycles	14	23.8 ± 37.9	14	23.8 ± 30.4		
4 cycles	11	9.0 ± 15.5	12	22.2 ± 32.8		
6 cycles	9	7.4 ± 14.6	11	24.2 ± 30.1	16.8 (-6.3 – 39.9)	0.144
P Baseline - 6 cycles		0.088		0.999		
Δ Baseline - 6 cycles	9	-25.9 ± 40.0	11	-0.0 ± 49.4	25.9 (-17.0 – 68.9)	0.221
Peripheral neuropathy						
Baseline	22	30.3 ± 32.3	23	17.3 ± 31.5	-12.9 (-32.1 – 6.3)	0.183
2 cycles	14	38.0 ± 38.9	13	17.9 ± 25.8		
4 cycles	11	42.4 ± 39.6	12	38.8 ± 39.7		
6 cycles	9	37.0 ± 42.3	11	48.4 ± 43.1	11.1 (-28.9 – 51.8)	0.559
P Baseline - 6 cycles		0.760		0.025		
Δ Baseline - 6 cycles	9	3.7 ± 35.1	11	36.3 ± 45.8	32.6 (-6.4 – 71.7)	0.096
Alopecia						
Baseline	22	31.8 ± 45.3	23	20.2 ± 31.3	-11.5 (-35.1 – 12.1)	0.330
2 cycles	14	61.9 ± 41.0	14	52.3 ± 44.7		
4 cycles	11	69.6 ± 34.8	12	63.8 ± 38.8		
6 cycles	9	62.9 ± 42.3	11	48.4 ± 45.6	-14.4 (-56.1 – 27.2)	0.475
P Baseline - 6 cycles		0.174		0.152		
Δ Baseline - 6 cycles	9	25.9 ± 52.1	11	24.2 ± 51.8	-1.6 (-50.7 – 47.3)	0.943

Pain in chest						
Baseline	22	37.8 ± 34.5	23	27.5 ± 29.5	-10.3 (-29.6 – 8.9)	0.286
2 cycles	14	33.3 ± 34.5	13	25.6 ± 30.8		
4 cycles	11	21.2 ± 22.4	12	33.3 ± 20.1		
6 cycles	9	22.2 ± 37.2	11	21.2 ± 22.4	-1.0 (-29.3 – 27.2)	0.941
P Baseline - 6 cycles		0.081		0.506		
Δ Baseline - 6 cycles	9	-11.1 ± 16.6	11	-6.0 ± 29.1	5.0 (-17.9 – 28.0)	0.651
Pain in arm or shoulder						
Baseline	22	33.3 ± 38.4	23	26.0 ± 28.3	-7.2 (-27.5 – 13.0)	0.475
2 cycles	14	33.3 ± 43.3	14	26.1 ± 35.0		
4 cycles	11	24.2 ± 21.5	12	27.7 ± 37.1		
6 cycles	9	33.3 ± 33.3	11	39.3 ± 29.1	6.0 (-23.2 – 35.3)	0.669
P Baseline - 6 cycles		0.195		0.341		
Δ Baseline - 6 cycles	9	11.1 ± 23.5	11	12.1 ± 40.2	1.0 (-30.9 – 32.9)	0.948
Pain in other parts						
Baseline	22	27.2 ± 19.6	23	23.1 ± 25.4	-4.0 (-17.8 – 9.6)	0.551
2 cycles	14	33.3 ± 29.2	14	21.4 ± 16.5		
4 cycles	11	21.2 ± 16.8	12	27.7 ± 23.9		
6 cycles	9	22.2 ± 16.6	11	30.3 ± 10.0	8.0 (-5.6 – 21.7)	0.225
P Baseline - 6 cycles		0.999		0.104		
Δ Baseline - 6 cycles	9	0 ± 23.5	11	12.1 ± 22.4	12.1 (-9.5 – 33.8)	0.256

Supplementary Table 5. Anxiety and Depression according to the HADS scale throughout study duration.

Health related quality of life	Baseline	2 cycles	p	4 cycles	p	6 cycles	p
Anxiety							
EPC	6 (4 – 9)	6 (2.5 – 9-)	0.825	6 (3 – 9)	0.559	7 (3 – 9.75)	0.744

C	7 (4 – 12)	6 (2.5 – 9)	0.031	6 (3 – 10)	0.578	6 (4 – 9)	0.437
Depression	0.086	0.494		0.539		0.763	
EPC	6 (3 – 9)	6 (2 – 8.25)	0.409	7 (5 – 9)	0.032	6 (3 – 9)	0.195
C	7 (4 – 10.2)	7 (4 – 10)	0.476	7 (5 – 10)	0.610	8 (3.2 – 10)	0.555
	0.046	0.150		0.857		0.173	
